# Health Correlates of Extended Longevity in Captive Ring‐Tailed Lemurs (*Lemur catta*)

**DOI:** 10.1002/ajp.70103

**Published:** 2025-12-11

**Authors:** Ruby L. Mustill, Laura N. Ellsaesser, Cathy V. Williams, Megan Petersdorf, Lydia K. Greene

**Affiliations:** ^1^ Interdisciplinary Doctoral Degree Program in Ecology and Evolutionary Biology Texas A&M University College Station Texas USA; ^2^ Department of Biology Texas A&M University College Station Texas USA; ^3^ The Duke Lemur Center Duke University Durham North Carolina USA; ^4^ Department of Anthropology Tulane University New Orleans Louisiana USA; ^5^ Department of Biology Duke University Durham North Carolina USA

**Keywords:** captivity, hematology, old age, serology, strepsirrhine

## Abstract

Captive primates maintained at accredited institutions can live extraordinarily long lives and, as a result, are useful models for understanding the physiology of aging. Many institutions monitor primate health using serum chemistry panels and complete blood counts (CBCs), assays that capture organ and immune function and provide rich data for retrospective research. In this study, we compiled results from 169 serum chemistry panels and 168 CBCs collected between 2011 and 2022 at the Duke Lemur Center from 60 ring‐tailed lemurs (*Lemur catta*), aged between 9 months and 32.8 years. Our dataset included 20 individuals who were 15 years or older, 10 of whom were 20 years or older. We found patterns consistent with gradual, age‐related change in biomarkers associated with pancreas, kidney, and hepatobiliary function. Whereas concentrations of some markers increased with increasing age (e.g., amylase, lipase, gamma‐glutamyl transferase, globulin, and total CO_2_), concentrations of others decreased with increasing age (e.g., total bilirubin, calcium, and anion gap). We found significant age‐by‐sex interaction effects on blood urea nitrogen and cholesterol values, with females exhibiting sharper age‐related increases in these analytes, particularly in late age, that could indicate steeper declines in kidney function than those experienced by males. Ultimately, our results capture a portrait of senescence in captive ring‐tailed lemurs with extended longevity, with implications for the management of geriatric lemurs under human care. More broadly, including lemurs with diverse social systems and ecologies in retrospective studies of aging could illuminate physiological trends deeply rooted in the primate family tree and those uniquely shaped by evolution in Madagascar.

## Summary

- We analyzed a dataset of serum chemistry panels and complete blood counts collected over 11 years from 60 ring‐tailed lemurs at the Duke Lemur Center.
- We found patterns consistent with gradual, age‐related change in markers associated with pancreas, kidney, and hepatobiliary function. Whereas concentrations of some biomarkers increased with increasing age (e.g., amylase, lipase, gamma‐glutamyl transferase, globulin, and total CO_2_), concentrations of others decreased with increasing age (e.g., total bilirubin, calcium, and anion gap).
- Age‐related variation in blood urea nitrogen, albumin, creatine kinase, and cholesterol was mediated by sex, such that males and females exhibited different patterns of change in these analytes across their lives.

## Graphical abstract

Male and female ring‐tailed lemurs exhibit distinct patterns of age‐related change in certain serum chemistry markers. Photo by David Haring.

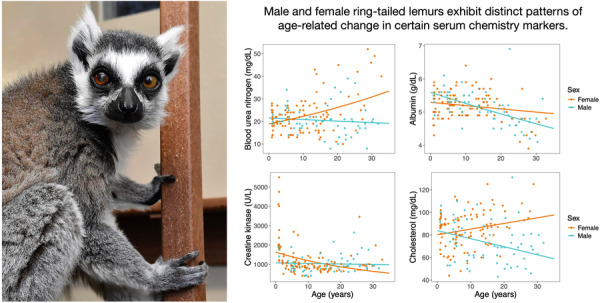

## 1 Introduction

Among mammals, primates are exceptionally long‐lived for their size and therefore present fascinating systems for understanding the physiology of aging (Charnov and Berrigan 1993; Jones 2011). In general, primate longevity can be linked to slow life history traits that include a prolonged juvenile period, later onset of reproductive maturity, lower fertility, and greater parental investment compared to other mammals (Charnov and Berrigan 1993; Jones 2011). Even in the wild, female primates of some species may live past reproductive senescence (Campos et al. 2022; Wood et al. 2023; Smit and Robbins 2025). For primate populations under human management, longevity can be further extended with readily available food and water, a lack of predation, and veterinary intervention. Indeed, there are reports of greatly extended lifespans in captive primates compared to their wild counterparts (Hill et al. 2001; Zehr et al. 2014; but see Tidière et al. 2016; Havercamp et al. 2019). Captive primates can therefore provide useful models for understanding the physiology of aging, with added relevance for human health (Smucny et al. 2001; Languille et al. 2012; Shively et al. 2021).

Studies of primate health across the lifespan, in both the wild and captivity, often rely on serological and hematological biomarkers assayed in standard serum chemistry panels and complete blood counts (CBCs). These panels give an overview of how an individual's organ and immune systems are functioning (Constable et al. 2017). By comparing an animal's serum chemistry and CBC results to reference ranges established for their species, veterinarians and researchers can confirm or rule out the presence of specific diseases, including those that are related to senescence (Tvedten and Thomas 2012; Constable et al. 2017). In many primates, serum chemistry and CBC values change with age (*Homo sapiens*: Bohnen et al. 1991; *Cebus apella*: Riviello and Wirz 2001; *Macaca mulatta*: Smucny et al. 2001; *Pan troglodytes*: Herndon and Tigges 2001; Videan et al. 2008; *Chlorocebus aethiops sabaeus*: Sato et al. 2005; *Propithecus diadema*: Irwin et al. 2010; *Microcebus murinus*: Marchal et al. 2012; *Macaca fascicularis*: Xie et al. 2013; *Macaca thibetana*: Wu et al. 2014; *Lemur catta*: Singleton et al. 2018). Some patterns are common across taxa. For example, blood urea nitrogen (BUN) generally increases with increasing age in diverse primates (Bohnen et al. 1991; Smucny et al. 2001; Videan et al. 2008; Marchal et al. 2012; Xie et al. 2013). Some patterns, however, differ between species. Serum creatinine concentrations, for instance, decrease with age in some primates (Smucny et al. 2001; Irwin et al. 2010), but increase with age in others (Videan et al. 2008; Marchal et al. 2012; Xie et al. 2013; Singleton et al. 2018). Importantly, variation in serum chemistry and CBC values across the lifespan is not necessarily the result of underlying disease, that is, of clinical concern, but may instead reflect normal changes that accompany the aging process (Bohnen et al. 1991; Smucny et al. 2001).

In this study, we add to this literature by examining age‐ and sex‐related variation in serum chemistry and CBC markers in a large, managed population of ring‐tailed lemurs (*Lemur catta*). Ring‐tailed lemurs are strepsirrhine primates endemic to southern Madagascar (Budnitz and Dainis 1975; Gould 2006). They are diurnal and omnivorous, and live in multi‐male, multi‐female groups (Sauther et al. 1999). Like other primates, ring‐tailed lemurs reach reproductive maturity later and live longer than similarly sized mammals (Richard et al. 2002). Among primates, however, lemurs fall on the fast end of the life history speed continuum (Kappeler 1996; Catlett et al. 2010). Moreover, female ring‐tailed lemurs are socially dominant over males (Jolly 1966; Richard 1987; Kappeler 1990) and have priority of access to resources (Jolly 1984; Parga and Thurau 2022), which makes them an interesting species for studying sex‐based differences in aging physiology.

To date, most existing serum chemistry and CBC data for ring‐tailed lemurs have come from studies of wild, free‐ranging populations in Madagascar (e.g., Dutton et al. 2003; Miller et al. 2007; Singleton et al. 2015, 2018). Age‐ and sex‐based differences in serum chemistry and CBC markers have been explicitly examined in a wild population from the Bezà Mahafaly Special Reserve in Madagascar (Singleton et al. 2018) and in a free‐ranging population introduced to St. Catherines Island, Georgia, USA (Page et al. 2024). In the wild, ring‐tailed lemurs are considered geriatric at 10 years (e.g., Cuozzo et al. 2010), and their maximum lifespan is around 15–20 years (Gould et al. 2003; Cuozzo et al. 2010; Ichino et al. 2015; Singleton et al. 2018). In the study conducted at Bezà Mahafaly, grouping the lemurs into three age categories—young (<5 years), adult (5–9.99 years), and old (≥10 years)—revealed that old animals had lower lymphocyte counts consistent with immunosenescence (Singleton et al. 2018). On St. Catherines Island, lemurs were differently categorized as infants (<1 year), juveniles (1–5.99 years), or adults (≥6 years), with a maximum age of 12 years. In this population, linear regressions indicated a positive relationship between age and markers like BUN, creatinine, lipase, GGT, globulin, and neutrophil count, and a negative relationship between age and markers like cholesterol, glucose, and calcium (Page et al. 2024). The differences between the results of these two studies could reflect variation in methodology, animal ages, or population biology.

**Figure 1:**
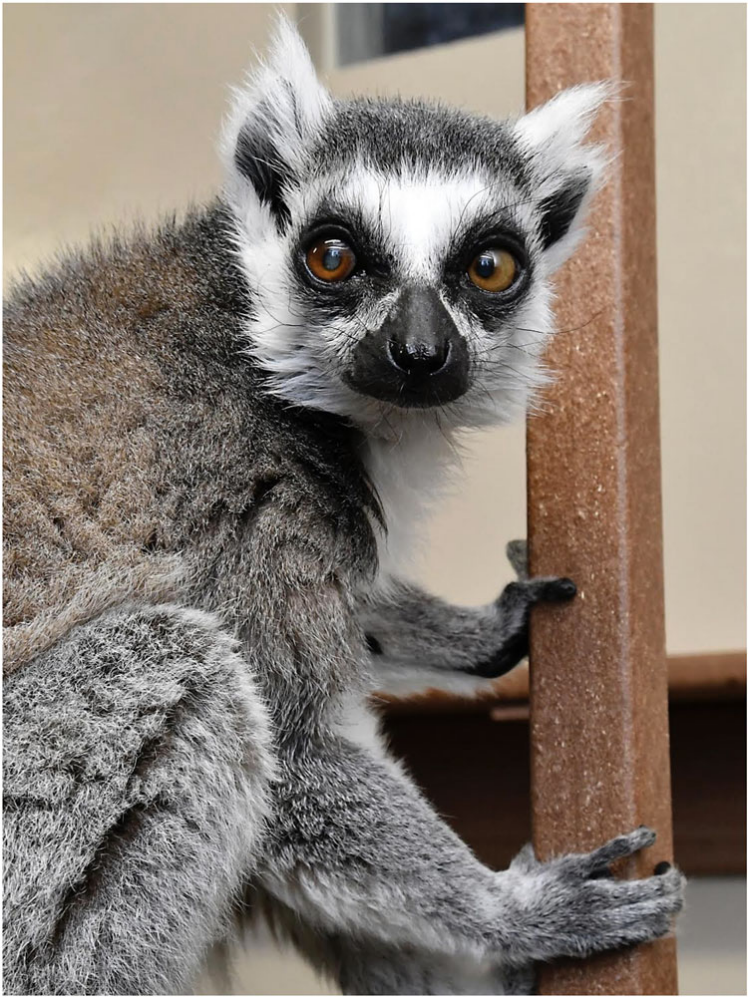
A geriatric lemur named Chloris, who lived to 34 years, 7 months, and 26 days and, in so doing, extended maximum longevity for her species in captivity at the Duke Lemur Center. Photo by David Haring.

Although a large number of captive ring‐tailed lemurs live in the United States (Dutton et al. 2003; Grogan et al. 2017), comparatively little is known about aging physiology in these human‐managed populations. Ring‐tailed lemurs are ecologically flexible (Sauther et al. 1999; Miller et al. 2007; LaFleur and Gould 2020) and adapt well to captive environments (Collins et al. 2017), where consistent access to food, water, and veterinary care seemingly increases their longevity. At the Duke Lemur Center, median longevity is 17.6 years for ring‐tailed lemurs who survive their first 30 days, and maximum lifespan is published at 32.7 years (Zehr et al. 2014). A more recent, unpublished case pushes the upper age limit for captive ring‐tailed lemurs to 34.6 years (Figure 1; authors' personal observations). We note that ring‐tailed lemurs at the Duke Lemur Center routinely live into their 20s and 30s, which is likely past reproductive senescence (i.e., cessation of reproduction) for female lemurs (Kappeler et al. 2022; Duke Lemur Center internal records). As captive primates age, their risk for developing several chronic conditions, like kidney disease, increases (Dutton et al. 2003; Miller et al. 2007; Shively et al. 2021). Understanding patterns of aging and indicators of age‐related disease is crucial to keeping captive primate populations well as they age under human management. The substantial number of ring‐tailed lemurs in captivity, and their extended lifespans, present an opportunity to study aging physiology in males and females; pinpoint markers that may reveal susceptibility to specific diseases in old age; and add comparative data to the literature, especially from old animals.

We therefore examined serum chemistry panels and CBCs collected over 11 years from the population of captive ring‐tailed lemurs at the Duke Lemur Center. Our aim was to determine the effects of age, sex, and the interaction between age and sex on serum chemistry and CBC markers, using an exploratory approach. Our research adds data from lemurs of advanced age (i.e., ≥20 years) to the published literature, which could support the diagnosis and treatment of illness in geriatric zoo lemurs by revealing which organ systems are most commonly affected by age and whether any consistent changes in healthspan are mediated by sex. By focusing specifically on captive lemurs, we work towards a deeper understanding of the physiological mechanisms responsible for extending longevity in captive primate populations.

## 2 Methods

### 2.1 Study Subjects

The subjects were 60 ring‐tailed lemurs (21 males; 39 females) housed at the Duke Lemur Center in Durham, NC, USA. At the Duke Lemur Center, all ring‐tailed lemurs are socially housed in standard indoor/outdoor enclosures. Some groups gain variable access to forest enclosures (0.4–6.5 ha) when overnight temperatures remain reliably above 3°C–5°C. Provisioned diets include primate chow and assorted fruits and vegetables, and lemurs granted forest access may additionally forage on local vegetation (Greene et al. 2024). Water is always freely available. All lemurs are individually recognizable via distinct features, colored collars, and unique tail shaves.

The subjects were maintained in accordance with the regulations set by the U.S. Department of Agriculture and the National Institutes of Health Guide for the Care and Use of Laboratory Animals. The protocols for this study adhered to the Principles for the Ethical Treatment of Non‐Human Primates established by the American Society of Primatologists and were approved by Duke University's Institutional Animal Care and Use Committee.

### 2.2 Biomedical Exams and Biological Sampling

At the Duke Lemur Center, lemurs receive routine biomedical exams throughout their lives from dedicated veterinary staff. Ring‐tailed lemurs typically receive their first exam around their first birthday; healthy adults receive full exams once every 2–4 years. In addition, healthy animals moving to other AZA‐accredited institutions receive pre‐shipment exams to confirm their health status. On routine and pre‐shipment exams, animals are carried in transport kennels to the veterinary clinic in the morning hours following an overnight fast. Following brief manual restraint, veterinary staff anesthetize lemurs (typically using dexmedetomidine: 0.02 mg/kg; midazolam: 0.2 mg/kg; supplemental sevoflurane as needed) and collect whole blood, usually from the saphenous or femoral vein. Aliquots of serum and whole blood are respectively submitted on the same day of sampling for serum chemistry panels and CBCs at IDEXX Laboratories (Louisville, KY, USA) and are kept refrigerated until shipment.

For this study, we selected results from serum chemistry panels and CBCs collected from ring‐tailed lemurs during health examinations between January 2011 and February 2022. The subjects ranged in age from 9 months to 32.8 years at the time of their exams. Most individuals were examined more than once during the 11‐year study period, and these animals are therefore represented in our dataset several times, at different ages. Of the 169 serum chemistry panels and 168 CBCs analyzed in our study, 60 of each were from 20 lemurs over 15 years old, and 37 of each were from 10 lemurs over 20 years old. Of these older animals, two had diagnosed chronic conditions, including one individual with type 2 diabetes and one individual with a pancreatic mass. We excluded panels from animals diagnosed with acute illness or disease at the time of sampling. Our final dataset includes 169 serum chemistry panels from 59 individuals (mean = 2.86 panels per individual; range: 1–8), and 168 CBCs from 60 individuals (mean = 2.80 panels per individual; range: 1–8). All markers measured were within the detectable range of laboratory equipment, although a few markers in a handful of panels were not recoverable due to laboratory error. As a result, the number of observations per analyte varies slightly. Our raw data are available in the supporting information.

### 2.3 Selected Markers

We selected for statistical analysis a subset of 21 markers from the serum chemistry panels and eight markers from the CBCs for which we had few to no missing data points. Because different axes of health can influence each of these markers, we provide a general, rather than comprehensive, overview of their use. For a more comprehensive review, see Thrall et al. (2012).

For the serum chemistry panels, we include BUN and creatinine as markers broadly indicative of kidney function. Whereas urea nitrogen is produced when the liver breaks down protein, creatinine is produced during muscle breakdown, and both are cleared by the kidneys (Hosten 1990). Creatinine may therefore also reflect muscle mass. We group the digestive enzymes, amylase and lipase, broadly as markers of pancreatic function and kidney clearance (but see Lam and Muniraj 2022 and Chatterjee et al. 2025 for other clinical implications of elevated serum amylase and lipase). Amylase breaks down carbohydrates and is produced both in the pancreas and in the salivary glands, whereas lipase breaks down fats and is produced primarily in the pancreas (Engelking 2015a, 2015b). We broadly group gamma‐glutamyl transferase (GGT), total bilirubin, alkaline phosphatase, alanine transaminase (ALT), and aspartate transaminase (AST) as hepatobiliary markers. The enzyme GGT signals cholestasis; bilirubin is produced during the breakdown of heme; alkaline phosphatase is a non‐specific indicator of cholestasis often induced by corticosteroids; and ALT and AST are liver enzymes, with the former being more liver specific than the latter (Johnston 1999; Giboney 2005). We use creatine kinase, an enzyme produced in response to tissue damage, as a marker of muscle injury or breakdown (Levine and Levine 2012). We also include cholesterol; glucose; the blood proteins albumin and globulin; and a suite of ions and electrolytes, namely sodium, potassium, chloride, calcium, phosphate, total CO_2_, and anion gap.

For the CBCs, we include markers of red blood cells, particularly total red blood cell counts; the protein hemoglobin (HBG); and hematocrit (HCT), that is, the volume percentage of red blood cells in blood. We also include markers of white blood cells, particularly total white blood cell counts and percentages of lymphocytes, monocytes, neutrophils, and eosinophils.

### 2.4 Statistical Analyses

For each biomarker, we visually inspected values to identify and exclude extreme outliers (see Tables S1 and S2). We ultimately excluded a single sample for each of three markers (ALT, AST, calcium) from different panels that seemingly reflect errors in data entry (i.e., repeated digits or missing decimal points). To be conservative, we also excluded one high BUN value, one high GGT value, and one high creatine kinase value, each from different panels. These values could be biologically real, but were above Duke Lemur Center reference intervals for ring‐tailed lemurs. We additionally excluded multiple outlying lipase values from the individual with a pancreatic mass, as well as multiple outlying cholesterol values from the individual with type 2 diabetes. For descriptive analyses, we determined ranges, means, and standard deviations for each marker, with outliers excluded (Tables 1 and 2). Summary statistics, *including* outlying datapoints, are reported in Table S2.

**Table 1:** Effects of lemur age and sex on serum chemistry marker values.

Analyte	Units	Range	Mean ± SD	Final model terms	Age		Sex (male)		Age ∗ Sex	
					*z*	*p*	*z*	*p*	*z*	*p*
Blood urea nitrogen (BUN)	mg/dL	8–52	22.1 ± 7.6	age ∗ sex	3.49	**< 0.001**	1.30	0.19	−2.85	**0.004**
Creatinine	mg/dL	0.5–1.5	0.8 ± 0.2	age + sex	1.19	0.24	−0.60	0.55		
Amylase	U/L	703–3743	2244.0 ± 552.2	age + sex	5.40	**< 0.001**	−0.86	0.39		
Lipase	U/L	8–476	63.4 ± 68.6	age + sex	3.30	**0.001**	0.11	0.91		
Gamma‐glutamyl transferase (GGT)	U/L	6–112	24.7 ± 15.7	age + sex	5.43	**< 0.001**	−2.76	**0.006**		
Total bilirubin	mg/dL	0.2–0.8	0.5 ± 0.1	age + sex	−3.17	**0.002**	1.36	0.17		
Alkaline phosphatase	U/L	24–439	181.2 ± 76.8	age + sex	0.58	0.56	−2.16	**0.031**		
Alanine transaminase (ALT)	U/L	27–562	104.1 ± 68.5	age + sex	1.22	0.22	−1.71	0.09		
Aspartate transaminase (AST)	U/L	8–125	27.9 ± 16.7	age + sex	−0.75	0.46	−1.69	0.09		
Albumin	g/dL	3.9–6.9	5.2 ± 0.5	age ∗ sex	−1.59	0.11	2.22	**0.026**	−2.47	**0.013**
Globulin	g/dL	0.4–2.3	1.3 ± 0.3	age + sex	4.84	**< 0.001**	−1.44	0.15		
Creatine kinase	U/L	357–5500	1172.1 ± 743.1	age ∗ sex	−4.50	**< 0.001**	−2.74	**0.006**	2.76	**0.006**
Cholesterol	mg/dL	43–131	80.8 ± 17.9	age ∗ sex	1.91	0.06	0.67	0.50	−3.19	**0.001**
Glucose	mg/dL	73–426	189.4 ± 69.8	age + sex	−0.44	0.66	−0.22	0.82		
Sodium (Na)	mmol/L	138–154	145.6 ± 2.6	age + sex	1.51	0.13	−0.12	0.91		
Potassium (K)	mmol/L	2.9–5.4	4.0 ± 0.4	age + sex	0.74	0.46	−1.17	0.24		
Chloride (Cl)	mmol/L	99–115	108.8 ± 2.7	age + sex	−0.22	0.83	−0.18	0.86		
Calcium (Ca)	mg/dL	7.9–11.1	9.4 ± 0.5	age + sex	−3.62	**< 0.001**	−0.45	0.65		
Phosphate	mg/dL	1.6–10.2	4.1 ± 1.4	age + sex	−0.83	0.40	−2.62	**0.009**		
Total CO_2_	mmol/L	10–32	22.6 ± 3.9	age + sex	4.24	**< 0.001**	−0.38	0.70		
Anion gap	mmol/L	10–29	18.3 ± 4.1	age + sex	−2.64	**0.008**	−0.35	0.73		

*Note:* Females are the reference group for the sex variable. Negative *z*‐values under the “Sex” column indicate that predicted marker values for males are lower than those for females. Statistically significant results (*p* ≤ 0.05) are in bold.

**Table 2:** Effects of lemur age and sex on CBC marker values.

Analyte	Units	Range	Mean ± SD	Final model terms	Age		Sex (male)	
					*z*	*p*	*z*	*p*
Red blood cell count	10^6^ cells/µL	6.1–11.0	7.4 ± 0.8	age + sex	3.17	**0.002**	1.54	0.12
Hemoglobin (HGB)	g/dL	12.2–20.7	14.8 ± 1.5	age + sex	2.94	**0.003**	1.26	0.21
Hematocrit (HCT)	%	40.4–73.2	54.3 ± 6.2	age + sex	−1.09	0.28	2.06	**0.040**
White blood cell count	cells/µL	3200–16200	7502.4 ± 2492.7	age + sex	−2.17	**0.030**	−2.38	**0.017**
Lymphocytes	%	10.0–85.0	43.3 ± 16.2	age + sex	−0.36	0.72	0.73	0.47
Monocytes	%	0.0–12.0	3.7 ± 2.3	age + sex	−0.60	0.55	−0.39	0.70
Neutrophils	%	8.0–86.0	50.0 ± 16.4	age + sex	0.06	0.95	−0.74	0.46
Eosinophils	%	0.0–17.0	2.8 ± 3.2	age + sex	1.28	0.20	0.24	0.81

*Note:* Females are the reference group for the sex variable. Negative *z*‐values under the “Sex” column indicate that predicted marker values for males are lower than those for females. Statistically significant results (*p* ≤ 0.05) are in bold.

To test the influence of lemur age and sex on health markers, we used a series of generalized linear mixed models in the glmmADMB package (version 0.8.3.3) (Fournier et al. 2012) in RStudio (version 2025.05.1 + 513; Posit Team 2025) and with R Software (version 4.5.1; R Core Team 2025). In each model, we included one serum chemistry or CBC marker as the response variable; lemur age (continuous, in years), sex (categorical: male or female), and the interaction between age and sex as fixed effects; and individual lemur identity as a random effect.

We first fitted each model with all three fixed effects: age, sex, and their interaction. If the interaction between age and sex was a significant predictor of variation in the response variable, we kept this largest model as our final model. If the interaction between age and sex was not significant, we removed the interaction from the model so that its presence would not prevent us from clearly interpreting the independent effects of age and sex. In these cases, our final models include only age and sex as fixed effects. Depending on the biomarker, we used Gaussian, gamma, and negative binomial distribution families. We used log link functions for the models fitted with gamma or negative binomial distributions. The results of our final models are presented in Tables 1 and 2. For the distribution families and link functions used in each model, see Tables S3 and S4. Estimates and standard errors are reported in Tables S5 and S6.

## 3 Results

### 3.1 Serum Chemistry Analytes

We found varying effects of lemur age, sex, and their interaction on serum chemistry analytes. For summary statistics and full statistical results, see Table 1 and Table S5. For summary statistics broken down by age categories, see Tables S8, S10, and S12.

Overall, age significantly predicted concentrations of 10 out of 21 total serum chemistry markers, with six having a positive association with age (BUN, amylase, lipase, GGT, globulin, and total CO_2_) and four having a negative association with age (total bilirubin, creatine kinase, calcium, and anion gap). Sex significantly predicted values of five serum chemistry markers (GGT, alkaline phosphatase, albumin, creatine kinase, and phosphate). The interaction between age and sex was significant for four serum chemistry markers (BUN, albumin, creatine kinase, and cholesterol). Seven serum chemistry markers did not vary significantly with age, sex, or their interaction (creatinine, ALT, AST, glucose, sodium, potassium, and chloride).

In three of the four models with a significant age‐by‐sex interaction effect, one or both main effects (i.e., age and sex) were also significant. However, the presence of the interaction in these models prevents us from being able to draw conclusions about the main effects independently. Thus, for each of these four markers—BUN, albumin, creatine kinase, and cholesterol—we conducted follow‐up analyses to examine the effects of age within each sex. We subsetted our data by sex and fitted separate generalized linear mixed models for males and females, with each analyte modeled as a function of age only, and with lemur identity included as a random term. Although the sex‐specific age slopes can be derived algebraically from the coefficients of the original interaction models, fitting the separate models allowed us to more clearly describe sex‐specific patterns of age‐related change in serum concentrations of BUN, albumin, creatine kinase, and cholesterol. The results of these models are presented in Table S7.

**Figure 2:**
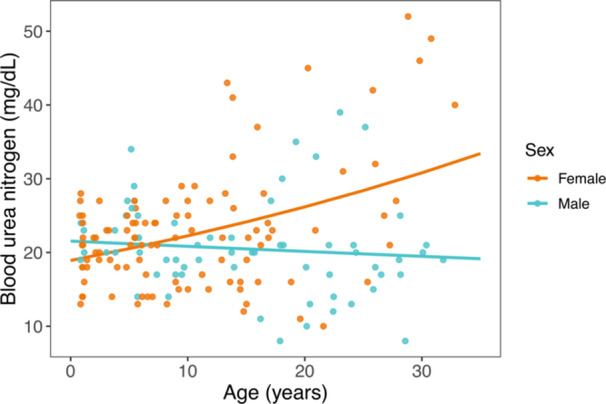
Serum concentrations of circulating blood urea nitrogen (BUN) relative to age for females (orange) and males (blue). Colored lines represent sex‐specific marginal predictions from generalized linear mixed models. Points represent raw data.

Examining our serum chemistry results by marker, we found that BUN concentrations (Figure 2) were significantly associated with the interaction between age and sex. Females exhibited a clear elevation in BUN with age, while males showed a slight decrease, if any change, with age. Creatinine values were not associated with age, sex, or their interaction.

**Figure 3:**
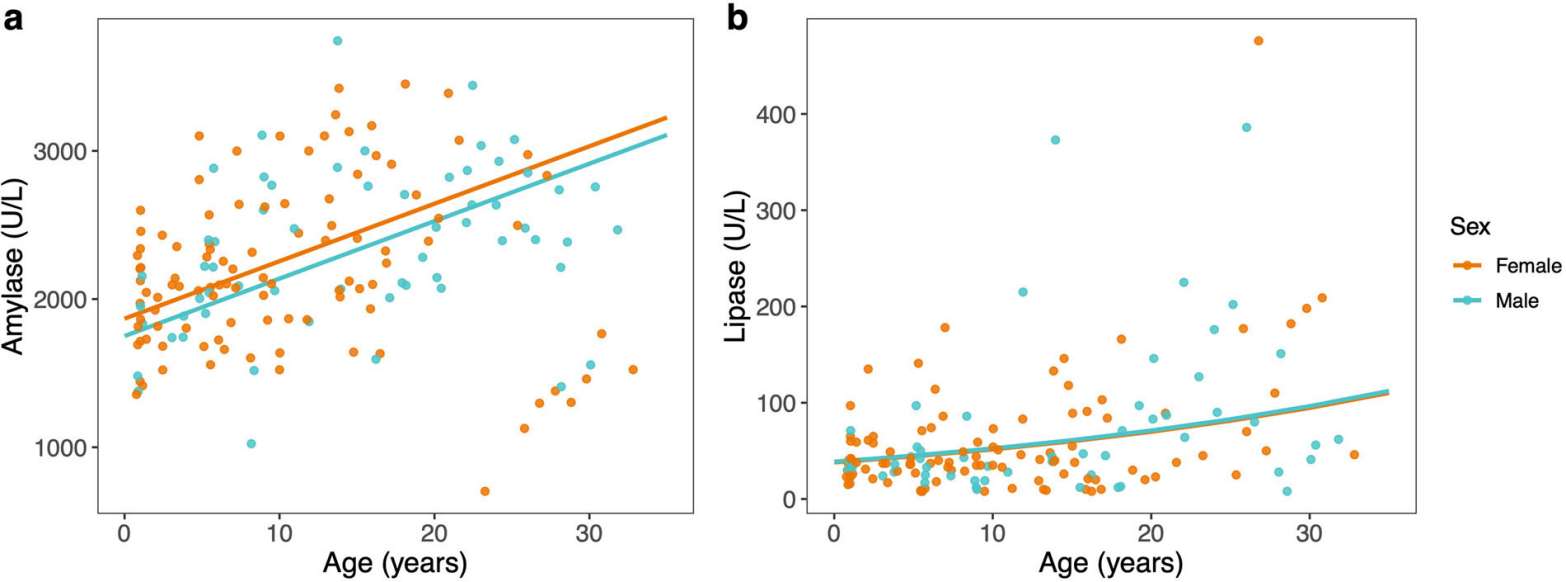
Serum concentrations of circulating (a) amylase and (b) lipase relative to age for females (orange) and males (blue). Colored lines represent sex‐specific marginal predictions from generalized linear mixed models. Points represent raw data.

**Figure 4:**
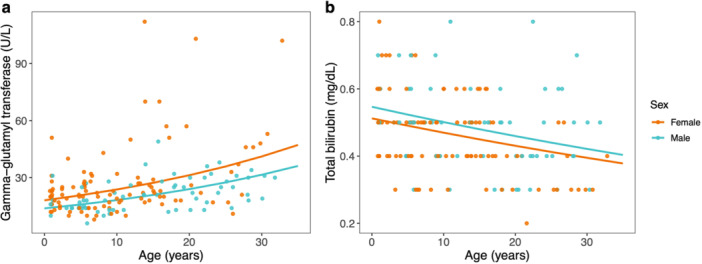
Serum concentrations of circulating (a) gamma‐glutamyl transferase (GGT) and (b) total bilirubin relative to age for females (orange) and males (blue). Colored lines represent sex‐specific marginal predictions from generalized linear mixed models. Points represent raw data.

Amylase (Figure 3a) and lipase (Figure 3b) increased with increasing age in both sexes and did not differ overall between sexes. GGT increased with increasing age in both sexes and was greater overall in females than in males (Figure 4a). Total bilirubin decreased with increasing age in both males and females and did not differ overall between sexes (Figure 4b). Alkaline phosphatase was greater in females than in males but did not vary with age. Neither ALT nor AST varied with age, sex, or their interaction.

**Figure 5:**
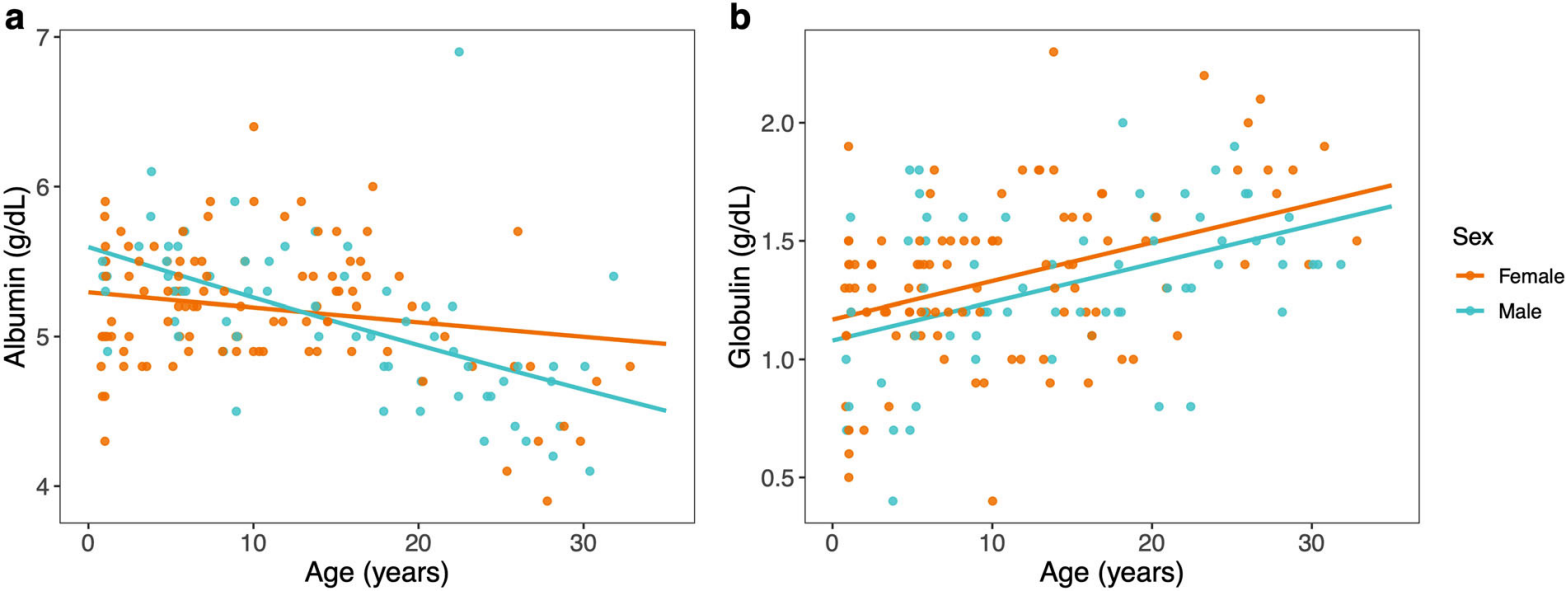
Serum concentrations of circulating (a) albumin and (b) globulin relative to age for females (orange) and males (blue). Colored lines represent sex‐specific marginal predictions from generalized linear mixed models. Points represent raw data.

Albumin concentrations were significantly predicted by the interaction between age and sex. Males exhibited a steeper decrease in albumin with increasing age, while females showed a more muted decrease with age (Figure 5a). In contrast, globulin was positively associated with age in both males and females (Figure 5b). Globulin concentrations did not vary by sex.

**Figure 6:**
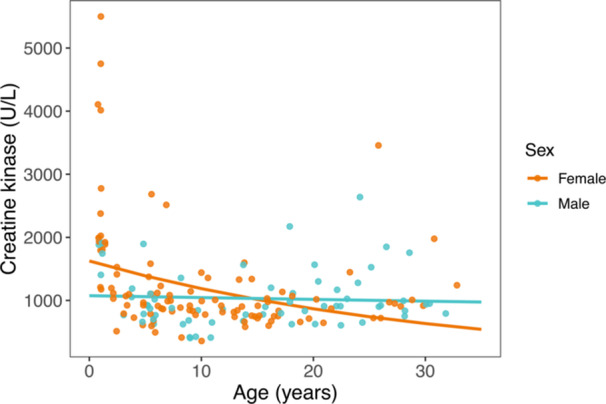
Serum concentrations of circulating creatine kinase relative to age for females (orange) and males (blue). Colored lines represent sex‐specific marginal predictions from generalized linear mixed models. Points represent raw data.

Creatine kinase was significantly associated with the interaction between age and sex, with females showing a clear and sharp decline in early life, and males largely showing stable values across the lifespan (Figure 6).

**Figure 7:**
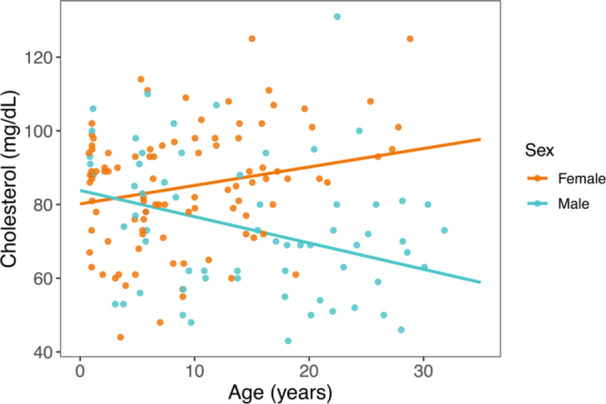
Serum concentrations of circulating cholesterol relative to age for females (orange) and males (blue). Colored lines represent sex‐specific marginal predictions from generalized linear mixed models. Points represent raw data.

Cholesterol levels were also significantly predicted by the interaction between age and sex. Specifically, cholesterol increased with increasing age in females but decreased with increasing age in males (Figure 7). We found no effect of age or sex on circulating glucose.

Of the ions and electrolytes, calcium, total CO_2_, and anion gap varied with age and not by sex. CO_2_ increased with age, whereas both calcium and anion gap decreased with age. Phosphate concentrations were greater in females than in males but did not vary with age. We found no significant effects of age or sex on sodium, potassium, or chloride.

### 3.2 CBC Analytes

We found varying effects of age and sex on CBC analytes. The interaction between age and sex was not significant in any of our largest models (see Methods), so we included only the two main effects in our final CBC models. For summary statistics and full statistical results, see Table 2 and Table S6. For summary statistics broken down by age categories, see Tables S9, S11, and S13.

**Figure 8:**
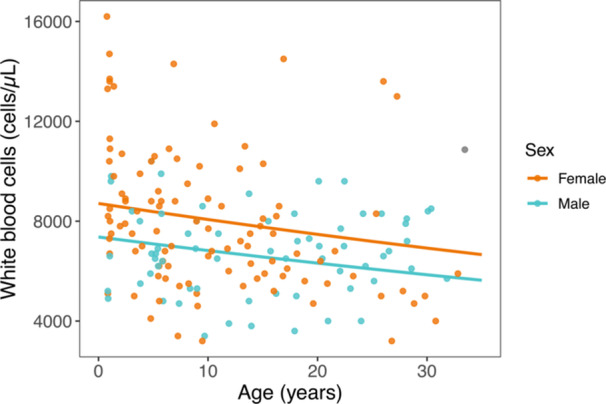
Circulating white blood cell counts relative to age for females (orange) and males (blue). Colored lines represent sex‐specific marginal predictions from generalized linear mixed models. Points represent raw data.

We found significant effects of age on three of eight total CBC markers, with two having a positive relationship with age (red blood cell count and hemoglobin) and one having a negative relationship with age (white blood cell count; Figure 8). We found significant effects of sex on two CBC markers (hematocrit and white blood cell count). Four CBC markers (percentages of lymphocytes, monocytes, neutrophils, and eosinophils) were not significantly associated with age or sex.

## 4 Discussion

By using a retrospective approach applied to a dataset of serological and hematological records collected from dozens of ring‐tailed lemurs in captivity over more than a decade, we identified clear patterns of age‐related changes in animal health. We observed significant relationships between age and amylase, lipase, GGT, and total bilirubin—markers that are variably associated with pancreas, kidney, and hepatobiliary function. We also found significant age‐by‐sex interactions for BUN and cholesterol, such that females showed greater values with increasing age relative to males. Creatine kinase, a marker of muscle damage or breakdown, was particularly elevated in some yearling females. This finding may indicate that the stress of first capture is more pronounced for juvenile females than for males, although perhaps ring‐tailed lemurs, like humans, simply exhibit elevated creatine kinase concentrations during early development (Fechner et al. 2024). Further sampling of young individuals, and males in particular, could prove clarifying. Finally, white blood cell counts decreased with increasing age in both sexes, likely reflecting immunosenescence. Taken together, our results help establish baseline health values for captive ring‐tailed lemurs throughout different life stages, including late age. Our results also highlight the value of captive primates for the study of aging and the role that longitudinal datasets can play in elucidating fine biological patterns.

One of the most striking findings of our study is that the majority of our aged lemur subjects were in good overall health. Wild ring‐tailed lemurs at the Bezà Mahafaly Special Reserve rarely live beyond 15 years (Singleton et al. 2018), whereas our dataset included 20 lemurs over 15 years of age, 10 of whom lived to be older than 20 years during the study period, and three of whom reached their 30s by the end of the study period (Figure 1). Of these older animals, only two had diagnosed chronic conditions that were being managed by veterinary staff. Many biomarkers in our panels were subject to age‐related changes that are consistent with gradual senescence, but not necessarily with consistent failure or acute decline of any particular organ system. This is in contrast to other lemur species that are susceptible to specific health conditions in captivity, including ruffed lemurs (*Varecia* spp.), which tend to accumulate hepatic iron (Williams et al. 2008) and have a high incidence of kidney disease in old age (Junge and Louis 2005), and sifakas (*Propithecus* spp.), which frequently succumb to infection and sepsis (Charles‐Smith et al. 2010; Cassady et al. 2018). Importantly, when we excluded animals with known chronic conditions, the serological and hematological values we analyzed generally fell within the Duke Lemur Center's internal reference intervals for adult ring‐tailed lemurs. In other words, our results might highlight age‐related trends in health markers in this species before they become of clinical concern.

When comparing our results to those previously published for other populations of ring‐tailed lemurs (Singleton et al. 2018; Page et al. 2024; see Tables S10–S13), we consistently find that white blood cells decrease with increasing age, which likely reflects gradual immunosenescence. We also find consistent trends between ring‐tailed lemurs from the Duke Lemur Center and those introduced to St. Catherines Island in markers such as BUN, lipase, GGT, and globulin, which increase with increasing age, and calcium, which decreases with increasing age (Page et al. 2024). Nevertheless, many of the other results we report differ from those published by Singleton et al. (2018) and Page et al. (2024), perhaps due, in part, to differences in methodology—but also due to the older ages of many animals included in our study. Notably, many of the oldest animals in these previous studies were between 10 and 15 years of age (Singleton et al. 2018; Page et al. 2024), whereas our study includes dozens of datapoints from animals aged between 15 and 32.8 years. Our finding of increasing amylase with increasing age, and the curious interaction effect between age and sex for BUN and cholesterol, could reflect age‐related changes in physiology that are only evident in ring‐tailed lemurs in later life or, for females, after reproductive senescence.

Our BUN and cholesterol results merit deeper investigation. Notably, females exhibited greater values of both markers with increasing age, while males showed, if anything, modest decreases. BUN is a nitrogenous product of protein metabolism related to kidney function or hydration status (Hosten 1990). High cholesterol can also be a risk factor for kidney disease (Schaeffner et al. 2003; Yamagata et al. 2007; but see Muntner et al. 2000). These markers may indicate that female ring‐tailed lemurs show greater signs of kidney decline as they age compared to males. This pattern is particularly curious because opposite trends are generally reported in other primate species, including chimpanzees and humans, where signs of kidney decline occur earlier and/or progress more quickly in males than in females (Bohnen et al. 1991; Neugarten et al. 2000; Herndon and Tigges 2001; Videan et al. 2008). One possible explanation for this pattern is that aging poses a particular challenge to female ring‐tailed lemurs because of the costs involved in maintaining social dominance. Female ring‐tailed lemurs are socially dominant over males (Jolly 1966; Richard 1987) and show strong but fluid hierarchies among females (Nakamichi and Koyama 1997; Nakamichi et al. 1997; Sauther et al. 1999). They maintain dominance through aggression (Kappeler 1990; Pereira et al. 1990), are hormonally “androgenized” during prenatal development (Drea 2011), and show significant circannual variation in adrenal hormones (von Engelhard et al. 2000; Drea 2007; Starling et al. 2010). In men, waning androgen concentrations with age correlate with declining kidney function (Kurita et al. 2016; van der Burgh et al. 2022). For female ring‐tailed lemurs, perhaps the physiological stress of maintaining dominance (Sapolsky 2005; Smith et al. 2016), including on the kidneys, leads to negative health outcomes that are only evident in aged females. In addition, the routine administration of hormonal contraceptives to female ring‐tailed lemurs in captivity may play a role in kidney health (Brändle et al. 1992; Monster 2001). Although we lacked data associated with dominance hierarchies and birth control administration, future studies could profitably ask how lifetime variation in rank, reproduction, and contraceptive use correlate with kidney function and health in female ring‐tailed lemurs across environments.

Regardless of how kidney function changes with age, captive ring‐tailed lemurs seem to have greater absolute BUN concentrations than do wild ring‐tailed lemurs (Dutton et al. 2003). Our average absolute BUN concentrations were 22.4 mg/dL ± 8.6 (mean ± SD), whereas averages reported for wild ring‐tailed lemurs are as low as 9 mg/dL ± 8 (Singleton et al. 2018) and 13.3 mg/dL ± 4.5 (Dutton et al. 2003). This pattern is also evident in ruffed lemurs (Junge and Louis 2005). In humans, protein‐rich diets can lead to elevated urea (Frank et al. 2009; Friedman et al. 2012; Ko et al. 2020). Wild ring‐tailed lemurs in Madagascar show seasonal variation in protein intake (Simmen et al. 2006), which is linked to gestation in females (Gould et al. 2011). There is also emerging evidence that lemurs, like other primates, evolved to sleuth out protein‐rich plants (Toda et al. 2021). In captivity, provisioned chows and orchard produce are high in protein (and sugar) relative to foraged wild plants (Greene et al. 2024). If captive lemurs consume more protein or experience less seasonality in protein intake than do their wild counterparts, this could explain population‐level differences in BUN and the subsequent stress placed on the kidneys. Although we cannot account for differences in methodology between studies, these results may call for a careful re‐evaluation of provisioned diets pertaining to crude and available protein.

Perhaps also related to dietary differences, we noted no change in the liver enzymes (ALT and AST) with age, which differs from patterns reported for some other primates (*Pan troglodytes*: Herndon and Tigges 2001; *Cebus apella*: Riviello and Wirz 2001; *Propithecus diadema*: Irwin et al. 2010; *Macaca fascicularis*: Xie et al. 2013; *M*. *thibetana*: Wu et al. 2014), including humans (Petroff et al. 2022). In Madagascar, ring‐tailed lemurs are omnivorous and naturally forage for an array of plant foods that are rich in condensed tannins and phenolics (Yamashita 2008; Gould et al. 2009). These compounds can require detoxification in the liver (Manach et al. 2004). That the chow and produce‐based diets fed to captive ring‐tailed lemurs are limited in plant secondary compounds (Greene et al. 2024) could perhaps extend liver health and function well into late life.

We focused our study on exploring age‐ and sex‐related variation in standard serological and hematological markers, but we emphasize that age and sex are not the only two variables that influence lemur health. We did not have access to more granular information about each individual's dietary, social, environmental, and birth control history—all factors that can influence health and healthspan. At the Duke Lemur Center specifically, some social groups of ring‐tailed lemurs gain access to multi‐acre forest enclosures, in which they may range and forage *ad libitum* during warmer months, with clear implications for naturalized behavior (Ganzhorn 1986; Greene et al. 2024). Emerging evidence in the Duke Lemur Center population points to greater lifetime stress—assessed by allostatic load—as correlated to smaller groups, more frequent group changes, and more time spent indoors (Seeley et al. 2021). Although these factors were beyond the scope of this paper, future studies could investigate how individual histories affect serum chemistry markers, CBC markers, and other health indicators in captive lemurs. We are also mindful that we included panels from two aged individuals after they were diagnosed with chronic conditions, and we emphasize that our study is descriptive rather than hypothesis‐driven. We analyzed one response variable at a time instead of adopting a multivariate approach; we did not include nonlinear predictors (e.g., splines) in models of potentially nonlinear relationships (such as the relationship between creatine kinase and age, Figure 6); and we applied no corrections for multiple comparisons. We posit that our results are likely to be most valuable in the context of captive management of ring‐tailed lemurs, adding to existing knowledge of ring‐tailed lemur biology and providing a foundation for the diagnosis of disease in geriatric animals in captivity. By demonstrating that extended longevity can coincide with extended healthspan in captive lemurs, we hope to encourage further research that can measure and promote the welfare of captive animals in old age.

The pronounced age‐related changes we observed in females highlight the importance of including sex as a variable in studies of animals—an idea that is gaining traction among biologists (Zucker and Beery 2010; Clayton and Collins 2014; Klein et al. 2015). To determine if the patterns we identified here are related to female dominance, future studies could examine age‐by‐sex interactions in markers of health in other female‐dominant taxa, including spotted hyenas (Frank 1986), meerkats (Clutton‐Brock et al. 2001), naked mole‐rats (Clarke and Faulkes 1997), and most other lemur species (Wright 1999). Researchers have devoted abundant attention to the relationships between sociality, stress, health, and aging in primates (e.g., Sapolsky 2005; Silk et al. 2010; Archie et al. 2014; Beehner and Bergman 2017; Brent et al. 2017; Thompson and Cords 2018; Campos et al. 2020; Snyder‐Mackler et al. 2020; Muller et al. 2021; Tung et al. 2023), but we advocate greater inclusion of lemurs into this conversation (e.g., Languille et al. 2012; Seeley et al. 2021). Beyond sex and sociality, lemurs provide an opportunity to examine aging in primates linked to neurodegeneration (Bons et al. 2006), telomere dynamics (Steinert et al. 2002), and torpor and hibernation (Blanco and Zehr 2015). More broadly, research on aging in lemurs with diverse ecologies, when placed in a comparative framework with haplorrhine primates, could elucidate aspects of aging physiology that are deeply rooted in primate history, as well as those that arose *de novo* in lemurs in response to the particular challenges of life in Madagascar (Wright 1999; Dewar and Richard 2007).

## Author Contributions

Ruby L. Mustill and Lydia K. Greene conceived of and designed the study. Ruby L. Mustill performed statistical analyses and data visualization with input from Lydia K. Greene and Megan Petersdorf. Ruby L. Mustill and Lydia K. Greene wrote the manuscript. Laura N. Ellsaesser and Cathy V. Williams contributed to data acquisition and results interpretation. All authors contributed to manuscript editing and preparation.

## Conflicts of Interest

The authors declare no conflicts of interest.

## Supporting information

Mustill‐et‐al‐supporting‐information.

Mustill‐et‐al‐serum‐data.

Mustill‐et‐al‐CBC‐data.

## Data Availability

The data that substantiate the findings of this study are available in the supporting information of this article.

## References

[ajp70103-bib-0001] Archie, E. A. , J. Tung , M. Clark , J. Altmann , and S. C. Alberts . 2014. “Social Affiliation Matters: Both Same‐Sex and Opposite‐Sex Relationships Predict Survival in Wild Female Baboons.” Proceedings of the Royal Society B: Biological Sciences 281, no. 1793: 20141261. 10.1098/rspb.2014.1261. PMC4173677 25209936

[ajp70103-bib-0002] Beehner, J. C. , and T. J. Bergman . 2017. “The Next Step for Stress Research in Primates: To Identify Relationships Between Glucocorticoid Secretion and Fitness.” Hormones and Behavior 91: 68–83. 10.1016/j.yhbeh.2017.03.003. 28284709

[ajp70103-bib-0003] Blanco, M. B. , and S. M. Zehr . 2015. “Striking Longevity in a Hibernating Lemur.” Journal of Zoology 296, no. 3: 177–188. 10.1111/jzo.12230.

[ajp70103-bib-0004] Bohnen, N. , C. P. Degenaar , and J. Jolles . 1991. “Influence of Age and Sex on 19 Blood Variables in Healthy Subjects.” Zeitschrift für Gerontologie 24: 339–345. 1441715

[ajp70103-bib-0005] Bons, N. , F. Rieger , D. Prudhomme , A. Fisher , and K. H. Krause . 2006. “ *Microcebus murinus*: A Useful Primate Model for Human Cerebral Aging and Alzheimer's Disease?” Genes, Brain and Behavior 5, no. 2: 120–130. 10.1111/j.1601-183X.2005.00149.x. 16507003

[ajp70103-bib-0006] Brändle, E. , E. Gottwald , H. Melzer , and H. G. Sieberth . 1992. “Influence of Oral Contraceptive Agents on Kidney Function and Protein Metabolism.” European Journal of Clinical Pharmacology 43: 643–646. 10.1007/BF02284965. 1493847

[ajp70103-bib-0007] Brent, L. J. N. , A. Ruiz‐Lambides , and M. L. Platt . 2017. “Family Network Size and Survival Across the Lifespan of Female Macaques.” Proceedings of the Royal Society B: Biological Sciences 284, no. 1854: 20170515. 10.1098/rspb.2017.0515. PMC5443955 28515205

[ajp70103-bib-0008] Budnitz, N. , and K. Dainis . 1975. “ *Lemur catta*: Ecology and Behavior.” In Lemur Biology, edited by I. Tattersall and R. W. Sussman , 219–235. Springer. 10.1007/978-1-4684-2121-7_12.

[ajp70103-bib-0009] van der Burgh, A. C. , S. R. Khan , S. J. C. M. M. Neggers , E. J. Hoorn , and L. Chaker . 2022. “The Role of Serum Testosterone and Dehydroepiandrosterone Sulfate in Kidney Function and Clinical Outcomes in Chronic Kidney Disease: A Systematic Review and Meta‐Analysis.” Endocrine Connections 11, no. 6: e220061. 10.1530/EC-22-0061. 35551117 PMC9254301

[ajp70103-bib-0010] Campos, F. A. , J. Altmann , M. Cords , et al. 2022. “Female Reproductive Aging in Seven Primate Species: Patterns and Consequences.” Proceedings of the National Academy of Sciences 119, no. 20: e2117669119. 10.1073/pnas.2117669119. PMC9171789 35533284

[ajp70103-bib-0011] Campos, F. A. , F. Villavicencio , E. A. Archie , F. Colchero , and S. C. Alberts . 2020. “Social Bonds, Social Status and Survival in Wild Baboons: A Tale of Two Sexes.” Philosophical Transactions of the Royal Society, B: Biological Sciences 375, no. 1811: 20190621. 10.1098/rstb.2019.0621. PMC7540948 32951552

[ajp70103-bib-0012] Cassady, K. , J. M. Cullen , and C. V. Williams . 2018. “Mortality in Coquerel's Sifakas (*Propithecus coquereli*) under Human Care: A Retrospective Survey From the Duke Lemur Center 1990–2015.” Journal of Zoo and Wildlife Medicine 49, no. 2: 315–323. 10.1638/2017-0242.1. 29900793

[ajp70103-bib-0013] Catlett, K. K. , G. T. Schwartz , L. R. Godfrey , and W. L. Jungers . 2010. “‘Life History Space’: A Multivariate Analysis of Life History Variation in Extant and Extinct Malagasy Lemurs.” American Journal of Physical Anthropology 142, no. 3: 391–404. 10.1002/ajpa.21236. 20091842

[ajp70103-bib-0014] Charles‐Smith, L. E. , P. Cowen , and R. Schopler . 2010. “Environmental and Physiological Factors Contributing to Outbreaks of *Cryptosporidium* in Coquerel's Sifaka (*Propithecus coquereli*) at the Duke Lemur Center: 1999–2007.” Journal of Zoo and Wildlife Medicine 41, no. 3: 438–444. 10.1638/2009-0160.1. 20945641

[ajp70103-bib-0015] Charnov, E. L. , and D. Berrigan . 1993. “Why Do Female Primates Have Such Long Lifespans and so Few Babies? Or Life in the Slow Lane.” Evolutionary Anthropology: Issues, News, and Reviews 1, no. 6: 191–194. 10.1002/evan.1360010604.

[ajp70103-bib-0016] Chatterjee, A. , R. Prado , H. Siddiki , and T. Stevens . 2025. “Clinical Evaluation of Patients With Elevated Serum Lipase.” Digestive Diseases and Sciences 70: 2264–2269. 10.1007/s10620-025-08929-9. 40119240 PMC12296881

[ajp70103-bib-0017] Clarke, F. M. , and C. G. Faulkes . 1997. “Dominance and Queen Succession in Captive Colonies of the Eusocial Naked Mole‐Rat, *Heterocephalus glaber* .” Proceedings. Biological Sciences 264, no. 1384: 993–1000. 10.1098/rspb.1997.0137. 9263466 PMC1688532

[ajp70103-bib-0018] Clayton, J. A. , and F. S. Collins . 2014. “Policy: NIH to Balance Sex in Cell and Animal Studies.” Nature 509, no. 7500: 282–283. 10.1038/509282a. 24834516 PMC5101948

[ajp70103-bib-0019] Clutton‐Brock, T. H. , P. N. M. Brotherton , A. F. Russell , et al. 2001. “Cooperation, Control, and Concession in Meerkat Groups.” Science 291, no. 5503: 478–481. 10.1126/science.291.5503.478. 11161200

[ajp70103-bib-0020] Collins, C. , I. Corkery , A. Haigh , S. McKeown , T. Quirke , and R. O'Riordan . 2017. “The Effects of Environmental and Visitor Variables on the Behavior of Free‐Ranging Ring‐Tailed Lemurs (*Lemur catta*) in Captivity.” Zoo Biology 36, no. 4: 250–260. 10.1002/zoo.21370. 28547779

[ajp70103-bib-0021] Constable, P. D. , K. W. Hinchcliff , S. H. Done , and W. Grünberg . 2017. “Clinical Examination and Making a Diagnosis.” In *Veterinary Medicine*: *A Textbook of the Diseases of Cattle*, *Horses*, *Sheep*, *Pigs*, *and Goats* (11th ed.), 1–28. Saunders.

[ajp70103-bib-0022] Cuozzo, F. P. , M. L. Sauther , L. Gould , R. W. Sussman , L. M. Villers , and C. Lent . 2010. “Variation in Dental Wear and Tooth Loss Among Known‐Aged, Older Ring‐Tailed Lemurs (*Lemur catta*): A Comparison Between Wild and Captive Individuals.” American Journal of Primatology 72, no. 11: 1026–1037. 10.1002/ajp.20846. 20872788

[ajp70103-bib-0023] Dewar, R. E. , and A. F. Richard . 2007. “Evolution in the Hypervariable Environment of Madagascar.” Proceedings of the National Academy of Sciences 104, no. 34: 13723–13727. 10.1073/pnas.0704346104. PMC1947998 17698810

[ajp70103-bib-0024] Drea, C. M. 2007. “Sex and Seasonal Differences in Aggression and Steroid Secretion in *Lemur catta*: Are Socially Dominant Females Hormonally ‘Masculinized’?” Hormones and Behavior 51, no. 4: 555–567. 10.1016/j.yhbeh.2007.02.006. 17382329

[ajp70103-bib-0025] Drea, C. M. 2011. “Endocrine Correlates of Pregnancy in the Ring‐Tailed Lemur (*Lemur catta*): Implications for the Masculinization of Daughters.” Hormones and Behavior 59, no. 4: 417–427. 10.1016/j.yhbeh.2010.09.011. 20932838

[ajp70103-bib-0026] Dutton, C. J. , R. E. Junge , and E. E. Louis . 2003. “Biomedical Evaluation of Free‐Ranging Ring‐Tailed Lemurs (*Lemur catta*) in Tsimanampetsotsa Strict Nature Reserve, Madagascar.” Journal of Zoo and Wildlife Medicine 34, no. 1: 16–24. 10.1638/1042-7260(2003)34[0016:BEOFRL]2.0.CO;2. 12723796

[ajp70103-bib-0027] von Engelhard, N. , P. M. Kappeler , and M. Heistermann . 2000. “Androgen Levels and Female Social Dominance in *Lemur catta* .” Proceedings of the Royal Society of London. Series B: Biological Sciences 267, no. 1452: 1533–1539. 10.1098/rspb.2000.1175. PMC1690709 11007329

[ajp70103-bib-0028] Engelking, L. R. 2015a. “Carbohydrate Digestion.” In Textbook of Veterinary Physiological Chemistry, 231–237. Elsevier. 10.1016/b978-0-12-391909-0.50038-4.

[ajp70103-bib-0029] Engelking, L. R. 2015b. “Lipid Digestion.” In Textbook of Veterinary Physiological Chemistry, 384–389. Elsevier. 10.1016/b978-0-12-391909-0.50060-8.

[ajp70103-bib-0114] Fechner, A ., A. Willenberg , N. Ziegelasch , A. Merkenschlager , W. Kiess , and M. Vogel . 2024. “Creatine Kinase Serum Levels in Children Revisited: New Reference Intervals From a Large Cohort of Healthy Children and Adolescents.” Clinica Chimica Acta 560: 119726. 10.1016/j.cca.2024.119726. 38735516

[ajp70103-bib-0030] Fournier, D. A. , H. J. Skaug , J. Ancheta , et al. 2012. “AD Model Builder: Using Automatic Differentiation for Statistical Inference of Highly Parameterized Complex Nonlinear Models.” Optimization Methods and Software 27, no. 2: 233–249. 10.1080/10556788.2011.597854.

[ajp70103-bib-0031] Frank, H. , J. Graf , U. Amann‐Gassner , et al. 2009. “Effect of Short‐Term High‐Protein Compared With Normal‐Protein Diets on Renal Hemodynamics and Associated Variables in Healthy Young Men.” American Journal of Clinical Nutrition 90, no. 6: 1509–1516. 10.3945/ajcn.2009.27601. 19812175

[ajp70103-bib-0032] Frank, L. G. 1986. “Social Organization of the Spotted Hyaena *Crocuta crocuta*. II. Dominance and Reproduction.” Animal Behaviour 34, no. 5: 1510–1527. 10.1016/S0003-3472(86)80221-4.

[ajp70103-bib-0033] Friedman, A. N. , L. G. Ogden , G. D. Foster , et al. 2012. “Comparative Effects of Low‐Carbohydrate High‐Protein Versus Low‐Fat Diets on the Kidney.” Clinical Journal of the American Society of Nephrology 7, no. 7: 1103–1111. 10.2215/CJN.11741111. 22653255 PMC3386674

[ajp70103-bib-0034] Ganzhorn, J. U. 1986. “Feeding Behavior of *Lemur catta* and *Lemur fulvus* .” International Journal of Primatology 7: 17–30. 10.1007/BF02692307.

[ajp70103-bib-0035] Giboney, P. T. 2005. “Mildly Elevated Liver Transaminase Levels in the Asymptomatic Patient.” American Family Physician 71, no. 6: 1105–1110. 15791889

[ajp70103-bib-0036] Gould, L. , P. Constabel , R. Mellway , and H. Rambeloarivony . 2009. “Condensed Tannin Intake in Spiny‐Forest‐Dwelling *Lemur catta* at Berenty Reserve, Madagascar, During Reproductive Periods.” Folia Primatologica 80, no. 4: 249–263. 10.1159/000252584. 19864917

[ajp70103-bib-0037] Gould, L. 2006. “ *Lemur catta* Ecology: What We Know and What We Need to Know.” In *Lemurs*: *Ecology and Adaptation* , edited by L. Gould and M. L. Sauther , 255–274. Springer. 10.1007/978-0-387-34586-4_12.

[ajp70103-bib-0038] Gould, L. , M. L. Power , N. Ellwanger , and H. Rambeloarivony . 2011. “Feeding Behavior and Nutrient Intake in Spiny Forest‐Dwelling Ring‐Tailed Lemurs (*Lemur catta*) During Early Gestation and Early to Mid‐Lactation Periods: Compensating in a Harsh Environment.” American Journal of Physical Anthropology 145, no. 3: 469–479. 10.1002/ajpa.21530. 21541932

[ajp70103-bib-0040] Gould, L. , R. W. Sussman , and M. L. Sauther . 2003. “Demographic and Life‐History Patterns in a Population of Ring‐Tailed Lemurs (*Lemur catta*) at Beza Mahafaly Reserve, Madagascar: A 15‐year Perspective.” American Journal of Physical Anthropology 120, no. 2: 182–194. 10.1002/ajpa.10151. 12541335

[ajp70103-bib-0041] Greene, L. K. , M. B. Blanco , C. Farmer , M. O'Malley , C. Gherardi , and M. T. Irwin . 2024. “Dietary and Nutritional Selections by Ecologically Diverse Lemurs in Nonnative Forests.” International Journal of Primatology 45: 947–950. 10.1007/s10764-024-00428-4.

[ajp70103-bib-0042] Grogan, K. E. , M. L. Sauther , F. P. Cuozzo , and C. M. Drea . 2017. “Genetic Wealth, Population Health: Major Histocompatibility Complex Variation in Captive and Wild Ring‐Tailed Lemurs (*Lemur catta*).” Ecology and Evolution 7, no. 19: 7638–7649. 10.1002/ece3.3317. 29043021 PMC5632616

[ajp70103-bib-0043] Havercamp, K. , K. Watanuki , M. Tomonaga , T. Matsuzawa , and S. Hirata . 2019. “Longevity and Mortality of Captive Chimpanzees in Japan From 1921 to 2018.” Primates 60: 525–535. 10.1007/s10329-019-00755-8. 31583490

[ajp70103-bib-0044] Herndon, J. G. , and J. Tigges . 2001. “Hematologic and Blood Biochemical Variables of Captive Chimpanzees: Cross‐Sectional and Longitudinal Analyses.” Comparative Medicine 51, no. 1: 60–69. 11926304

[ajp70103-bib-0045] Hill, K. , C. Boesch , J. Goodall , A. Pusey , J. Williams , and R. Wrangham . 2001. “Mortality Rates Among Wild Chimpanzees.” Journal of Human Evolution 40, no. 5: 437–450. 10.1006/jhev.2001.0469. 11322804

[ajp70103-bib-0046] Hosten, A. O. 1990. “Chapter 193: BUN and Creatinine.” In *Clinical Methods*: *The History*, *Physical*, *and Laboratory Examinations* , edited by H. K. Walker , W. D. Hall , and J. W. Hurst , (3rd ed.). Butterworths. https://www.ncbi.nlm.nih.gov/books/NBK305/. 21250045

[ajp70103-bib-0047] Ichino, S. , T. Soma , N. Miyamoto , et al. 2015. “Lifespan and Reproductive Senescence in a Free‐Ranging Ring‐Tailed Lemur (*Lemur catta*) Population at Berenty, Madagascar.” Folia Primatologica 86, no. 1–2: 134–139. 10.1159/000368670. 26022309

[ajp70103-bib-0048] Irwin, M. T. , R. E. Junge , J. L. Raharison , and K. E. Samonds . 2010. “Variation in Physiological Health of Diademed Sifakas Across Intact and Fragmented Forest at Tsinjoarivo, Eastern Madagascar.” American Journal of Primatology 72, no. 11: 1013–1025. 10.1002/ajp.20847. 20872787

[ajp70103-bib-0049] Johnston, D. E. 1999. “Special Considerations in Interpreting Liver Function Tests.” American Family Physician 59, no. 8: 2223–2230. 10221307

[ajp70103-bib-0050] Jolly, A. 1966. Lemur Behavior. University of Chicago Press.

[ajp70103-bib-0051] Jolly, A. 1984. “The Puzzle of Female Feeding Priority.” In *Female Primates*: *Studies by Women Primatologists* , edited by M. F. Small , 197–215. Liss.

[ajp70103-bib-0052] Jones, J. H. 2011. “Primates and the Evolution of Long, Slow Life Histories.” Current biology: CB 21, no. 18: 708–717. 10.1016/j.cub.2011.08.025. PMC3192902 21959161

[ajp70103-bib-0053] Junge, R. E. , and E. E. Louis . 2005. “Preliminary Biomedical Evaluation of Wild Ruffed Lemurs (*Varecia variegata* and *V*. *rubra*).” American Journal of Primatology 66, no. 1: 85–94. 10.1002/ajp.20129. 15898067

[ajp70103-bib-0054] Kappeler, P. M. 1990. “Female Dominance in *Lemur catta*: More Than Just Female Feeding Priority.” Folia Primatologica; International Journal of Primatology 55, no. 2: 92–95. 10.1159/000156504. 2227726

[ajp70103-bib-0055] Kappeler, P. M. 1996. “Causes and Consequences of Life‐History Variation Among Strepsirhine Primates.” The American Naturalist 148, no. 5: 868–891. 10.1086/285960.

[ajp70103-bib-0056] Kappeler, P. M. , L. Pethig , L. Prox , and C. Fichtel . 2022. “Reproductive Senescence in Two Lemur Lineages.” Frontiers in Ecology and Evolution 10: 894344. 10.3389/fevo.2022.894344.

[ajp70103-bib-0057] Klein, S. L. , L. Schiebinger , M. L. Stefanick , et al. 2015. “Sex Inclusion in Basic Research Drives Discovery.” Proceedings of the National Academy of Sciences 112, no. 17: 5257–5258. 10.1073/pnas.1502843112. PMC4418862 25902532

[ajp70103-bib-0058] Ko, G. J. , C. M. Rhee , K. Kalantar‐Zadeh , and S. Joshi . 2020. “The Effects of High‐Protein Diets on Kidney Health and Longevity.” Journal of the American Society of Nephrology 31, no. 8: 1667–1679. 10.1681/ASN.2020010028. 32669325 PMC7460905

[ajp70103-bib-0059] Kurita, N. , S. Horie , S. Yamazaki , et al. 2016. “Low Testosterone Levels and Reduced Kidney Function in Japanese Adult Men: The Locomotive Syndrome and Health Outcome in Aizu Cohort Study.” Journal of the American Medical Directors Association 17, no. 4: 371.e1–371.e6. 10.1016/j.jamda.2016.01.011. 26926336

[ajp70103-bib-0060] LaFleur, M. , and L. Gould . 2020. *Lemur catta*. The IUCN Red List of Threatened Species 2020: e.T11496A115565760. 10.2305/IUCN.UK.2020-2.RLTS.T11496A115565760.en.

[ajp70103-bib-0061] Lam, R. , and T. Muniraj . 2022. “Hyperamylasemia.” In StatPearls. StatPearls Publishing. https://www.ncbi.nlm.nih.gov/books/NBK559273/. 32644699

[ajp70103-bib-0062] Languille, S. , S. Blanc , O. Blin , et al. 2012. “The Grey Mouse Lemur: A Non‐Human Primate Model for Ageing Studies.” Ageing Research Reviews 11, no. 1: 150–162. 10.1016/j.arr.2011.07.001. 21802530

[ajp70103-bib-0063] Levine, J. M. , and G. J. Levine . 2012. “Neurologic Disorders.” In Small Animal Clinical Diagnosis by Laboratory Methods, edited by M. D. Willard and H. Tvedten , 304–314. Elsevier. 10.1016/b978-1-4377-0657-4.00014-4.

[ajp70103-bib-0064] Manach, C. , A. Scalbert , C. Morand , C. Rémésy , and L. Jiménez . 2004. “Polyphenols: Food Sources and Bioavailability.” The American Journal of Clinical Nutrition 79, no. 5: 727–747. 10.1093/ajcn/79.5.727. 15113710

[ajp70103-bib-0065] Marchal, J. , O. Dorieux , L. Haro , F. Aujard , and M. Perret . 2012. “Characterization of Blood Biochemical Markers During Aging in the Grey Mouse Lemur (*Microcebus murinus*): Impact of Gender and Season.” BMC Veterinary Research 8: 211. 10.1186/1746-6148-8-211. 23131178 PMC3514280

[ajp70103-bib-0066] Miller, D. S. , M. L. Sauther , M. Hunter‐Ishikawa , et al. 2007. “Biomedical Evaluation of Free‐Ranging Ring‐Tailed Lemurs (*Lemur catta*) in Three Habitats at the Beza Mahafaly Special Reserve, Madagascar.” Journal of Zoo and Wildlife Medicine 38, no. 2: 201–216. 10.1638/1042-7260(2007)038[0201:BEOFRL]2.0.CO;2. 17679503

[ajp70103-bib-0067] Monster, T. B. M. 2001. “Oral Contraceptive Use and Hormone Replacement Therapy Are Associated With Microalbuminuria.” Archives of Internal Medicine 161, no. 16: 2000–2005. 10.1001/archinte.161.16.2000. 11525702

[ajp70103-bib-0068] Muller, M. N. , D. K. Enigk , S. A. Fox , et al. 2021. “Aggression, Glucocorticoids, and the Chronic Costs of Status Competition for Wild Male Chimpanzees.” Hormones and Behavior 130: 104965. 10.1016/j.yhbeh.2021.104965. 33676127 PMC8043126

[ajp70103-bib-0069] Muntner, P. , J. Coresh , J. C. Smith , J. Eckfeldt , and M. J. Klag . 2000. “Plasma Lipids and Risk of Developing Renal Dysfunction: The Atherosclerosis Risk in Communities Study.” Kidney International 58, no. 1: 293–301. 10.1046/j.1523-1755.2000.00165.x. 10886574

[ajp70103-bib-0070] Nakamichi, M. , and N. Koyama . 1997. “Social Relationships Among Ring‐Tailed Lemurs (*Lemur catta*) in Two Free‐Ranging Troops at Berenty Reserve, Madagascar.” International Journal of Primatology 18: 73–93. 10.1023/A:1026393223883.

[ajp70103-bib-0071] Nakamichi, M. , M. L. O. Rakototiana , and N. Koyama . 1997. “Effects of Spatial Proximity and Alliances on Dominance Relations Among Female Ring‐Tailed Lemurs (*Lemur catta*) at Berenty Reserve, Madagascar.” Primates 38: 331–340. 10.1007/BF02381620.

[ajp70103-bib-0072] Neugarten, J. , A. Acharya , and S. R. Silbiger . 2000. “Effect of Gender on the Progression of Nondiabetic Renal Disease: A Meta‐Analysis.” Journal of the American Society of Nephrology 11, no. 2: 319–329. 10.1681/ASN.V112319. 10665939

[ajp70103-bib-0073] Page, A. , D. Brenner , and T. M. Norton . 2024. “Retrospective Hematology and Serum Biochemistry of Ring‐Tailed Lemurs (*Lemur catta*) on St. Catherines Island, Georgia, USA.” Journal of Zoo and Wildlife Medicine 55, no. 2: 436–446. 10.1638/2022-0088. 38875200

[ajp70103-bib-0074] Parga, J. A. , and E. Thurau . 2022. “Food Availability and Male Deference in the Female‐Dominant Ring‐Tailed Lemur, *Lemur catta* .” American Journal of Primatology 84, no. 9: e23422. 10.1002/ajp.23422. 35860858 PMC9539500

[ajp70103-bib-0075] Pereira, M. E. , R. Kaufman , P. M. Kappeler , and D. J. Overdorff . 1990. “Female Dominance Does Not Characterize All of the *Lemuridae* .” Folia Primatologica 55: 96–103. 10.1159/000156505. 2227727

[ajp70103-bib-0076] Petroff, D. , O. Bätz , K. Jedrysiak , J. Kramer , T. Berg , and J. Wiegand . 2022. “Age Dependence of Liver Enzymes: An Analysis of over 1,300,000 Consecutive Blood Samples.” Clinical Gastroenterology and Hepatology 20, no. 3: 641–650. 10.1016/j.cgh.2021.01.039. 33524594

[ajp70103-bib-0077] Posit Team . 2025. RStudio: Integrated Development Environment for R. Posit Software, PBC, Boston, MA. https://posit.co/.

[ajp70103-bib-0078] R Core Team . 2025. R: A Language and Environment for Statistical Computing. R Foundation for Statistical Computing, Vienna, Austria. https://www.R-project.org/.

[ajp70103-bib-0079] Richard, A. F. , R. E. Dewar , M. Schwartz , and J. Ratsirarson . 2002. “Life in the Slow Lane? Demography and Life Histories of Male and Female Sifaka (*Propithecus verreauxi verreauxi*).” Journal of Zoology 256, no. 4: 421–436. 10.1017/S0952836902000468.

[ajp70103-bib-0080] Richard, A. F. 1987. “Malagasy Prosimians: Female Dominance.” In Primate Societies, edited by B. B. Smuts , D. L. Cheney , R. M. Seyfarth , R. W. Wrangham , and T. T. Struhsaker , 25–33. University of Chicago Press.

[ajp70103-bib-0081] Riviello, M. C. , and A. Wirz . 2001. “Haematology and Blood Chemistry of *Cebus apella* in Relation to Sex and Age.” Journal of Medical Primatology 30, no. 6: 308–312. 10.1034/j.1600-0684.2001.300604.x. 11990530

[ajp70103-bib-0082] Sapolsky, R. M. 2005. “The Influence of Social Hierarchy on Primate Health.” Science 308, no. 5722: 648–652. 10.1126/science.1106477. 15860617

[ajp70103-bib-0083] Sato, A. , L. A. Fairbanks , T. Lawson , and G. W. Lawson . 2005. “Effects of Age and Sex on Hematologic and Serum Biochemical Values of Vervet Monkeys (*Chlorocebus aethiops sabaeus*).” Contemporary Topics in Laboratory Animal Science 44, no. 1: 29–34. 15697196

[ajp70103-bib-0084] Sauther, M. L. , R. W. Sussman , and L. Gould . 1999. “The Socioecology of the Ringtailed Lemur: Thirty‐Five Years of Research.” Evolutionary Anthropology: Issues, News, and Reviews 8, no. 4: 120–132. 10.1002/(SICI)1520-6505(1999)8:4<120::AID-EVAN3>3.0.CO;2-O.

[ajp70103-bib-0085] Schaeffner, E. S. , T. Kurth , G. C. Curhan , et al. 2003. “Cholesterol and the Risk of Renal Dysfunction in Apparently Healthy Men.” Journal of the American Society of Nephrology 14, no. 8: 2084–2091. 10.1681/ASN.V1482084. 12874462

[ajp70103-bib-0086] Seeley, K. E. , K. L. Proudfoot , B. Wolfe , and D. E. Crews . 2021. “Assessing Allostatic Load in Ring‐Tailed Lemurs (*Lemur catta*).” Animals: An Open Access Journal from MDPI 11, no. 11: 3074. 10.3390/ani11113074. 34827806 PMC8614249

[ajp70103-bib-0087] Shively, C. A. , A. Lacreuse , B. M. Frye , E. S. Rothwell , and M. Moro . 2021. “Nonhuman Primates at the Intersection of Aging Biology, Chronic Disease, and Health: An Introduction to the *American Journal of Primatology* Special Issue on Aging, Cognitive Decline, and Neuropathology in Nonhuman Primates.” American Journal of Primatology 83, no. 11: e23309. 10.1002/ajp.23309. 34403529 PMC8935964

[ajp70103-bib-0088] Silk, J. B. , J. C. Beehner , T. J. Bergman , et al. 2010. “Strong and Consistent Social Bonds Enhance the Longevity of Female Baboons.” Current Biology 20, no. 15: 1359–1361. 10.1016/j.cub.2010.05.067. 20598541

[ajp70103-bib-0089] Simmen, B. , M. L. Sauther , T. Soma , et al. 2006. “Plant Species Fed on by *Lemur catta* in Gallery Forests of the Southern Domain of Madagascar.” In *Ringtailed Lemur Biology*: Lemur catta *in Madagascar* , edited by A. Jolly , R. W. Sussman , N. Koyama , H. Rasamimanana , 55–68. Springer. 10.1007/978-0-387-34126-2.

[ajp70103-bib-0090] Singleton, C. L. , A. M. Norris , M. L. Sauther , F. P. Cuozzo , and I. A. Y. Jacky . 2015. “Ring‐Tailed Lemur (Lemur catta) Health Parameters Across Two Habitats With Varied Levels of Human Disturbance at the Bezà Mahafaly Special Reserve, Madagascar.” Folia Primatologica 86, no. 1–2: 56–65. 10.1159/000369554. 26022301

[ajp70103-bib-0091] Singleton, C. L. , M. L. Sauther , F. P. Cuozzo , and I. A. Y. Jacky . 2018. “Age‐Related Changes in Hematology and Blood Biochemistry Values in Endangered, Wild Ring‐Tailed Lemurs (Lemur catta) at the Bezà Mahafaly Special Reserve, Madagascar.” Journal of Zoo and Wildlife Medicine 49, no. 1: 30–47. 10.1638/2017-0008R1.1. 29517441

[ajp70103-bib-0092] Smit, N. , and M. M. Robbins . 2025. “Post‐Reproductive Lifespan in Wild Mountain Gorillas.” Proceedings of the National Academy of Sciences 122, no. 42: e2510998122. 10.1073/pnas.2510998122. PMC12557517 41082668

[ajp70103-bib-0093] Smith, T. E. , C. M. McCusker , J. M. G. Stevens , and R. W. Elwood . 2016. “Patterns of Behaviour, Group Structure and Reproductive Status Predict Levels of Glucocorticoid Metabolites in Zoo‐Housed Ring‐Tailed Lemurs, Lemur Catta.” Folia Primatologica 86, no. 6: 506–524. 10.1159/000442587. 26824528

[ajp70103-bib-0094] Smucny, D. A. , D. B. Allison , D. K. Ingram , et al. 2001. “Changes in Blood Chemistry and Hematology Variables During Aging in Captive Rhesus Macaques (Macaca Mulatta).” Journal of Medical Primatology 30, no. 3: 161–173. 10.1111/j.1600-0684.2001.tb00005.x. 11515672

[ajp70103-bib-0095] Snyder‐Mackler, N. , J. R. Burger , L. Gaydosh , et al. 2020. “Social Determinants of Health and Survival in Humans and Other Animals.” Science 368, no. 6493: eaax9553. 10.1126/science.aax9553. 32439765 PMC7398600

[ajp70103-bib-0096] Starling, A. P. , M. J. E. Charpentier , C. Fitzpatrick , E. S. Scordato , and C. M. Drea . 2010. “Seasonality, Sociality, and Reproduction: Long‐Term Stressors of Ring‐Tailed Lemurs (*Lemur catta*).” Hormones and Behavior 57, no. 1: 76–85. 10.1016/j.yhbeh.2009.09.016. 19804779

[ajp70103-bib-0097] Steinert, S. , D. M. White , Y. Zou , J. W. Shay , and W. E. Wright . 2002. “Telomere Biology and Cellular Aging in Nonhuman Primate Cells.” Experimental Cell Research 272, no. 2: 146–152. 10.1006/excr.2001.5409. 11777339

[ajp70103-bib-0098] Thompson, N. A. , and M. Cords . 2018. “Stronger Social Bonds Do Not Always Predict Greater Longevity in a Gregarious Primate.” Ecology and Evolution 8, no. 3: 1604–1614. 10.1002/ece3.3781. 29435236 PMC5792528

[ajp70103-bib-0099] Thrall, M. A. , Weiser, G. , Allison, R. W. , and Campbell, T. W. , eds. 2012. Veterinary Hematology and Clinical Chemistry. John Wiley & Sons.

[ajp70103-bib-0100] Tidière, M. , J. M. Gaillard , V. Berger , et al. 2016. “Comparative Analyses of Longevity and Senescence Reveal Variable Survival Benefits of Living in Zoos Across Mammals.” Scientific Reports 6: 36361. 10.1038/srep36361. 27819303 PMC5098244

[ajp70103-bib-0101] Toda, Y. , T. Hayakawa , A. Itoigawa , et al. 2021. “Evolution of the Primate Glutamate Taste Sensor From a Nucleotide Sensor.” Current Biology 31, no. 20: 4641–4649.e5. 10.1016/j.cub.2021.08.002. 34450087

[ajp70103-bib-0102] Tung, J. , E. C. Lange , S. C. Alberts , and E. A. Archie . 2023. “Social and Early Life Determinants of Survival From Cradle to Grave: A Case Study in Wild Baboons.” Neuroscience and Biobehavioral Reviews 152: 105282. 10.1016/j.neubiorev.2023.105282. 37321362 PMC10529797

[ajp70103-bib-0103] Tvedten, H. , and J. S. Thomas . 2012. “General Laboratory Concepts.” In Small Animal Clinical Diagnosis by Laboratory Methods, edited by M. D. Willard and H. Tvedten , 1–11. Elsevier. 10.1016/B978-1-4377-0657-4.00001-6/.

[ajp70103-bib-0104] Videan, E. N. , J. Fritz , and J. Murphy . 2008. “Effects of Aging on Hematology and Serum Clinical Chemistry in Chimpanzees (*Pan troglodytes*).” American Journal of Primatology 70, no. 4: 327–338. 10.1002/ajp.20494. 17943981

[ajp70103-bib-0105] Williams, C. V. , R. E. Junge , and I. H. Stalis . 2008. “Evaluation of Iron Status in Lemurs by Analysis of Serum Iron and Ferritin Concentrations, Total Iron‐Binding Capacity, and Transferrin Saturation.” Journal of the American Veterinary Medical Association 232, no. 4: 578–585. 10.2460/javma.232.4.578. 18279098

[ajp70103-bib-0106] Wood, B. M. , J. D. Negrey , J. L. Brown , et al. 2023. “Demographic and Hormonal Evidence for Menopause in Wild Chimpanzees.” Science 382, no. 6669: 5473. 10.1126/science.add5473. PMC10645439 37883540

[ajp70103-bib-0107] Wright, P. C. 1999. “Lemur Traits and Madagascar Ecology: Coping With an Island Environment.” American Journal of Physical Anthropology 110: 31–72. 10.1002/(SICI)1096-8644(1999)110:29+<31::AID-AJPA3>3.0.CO;2-0. 10601983

[ajp70103-bib-0108] Wu, D. , Y. Yi , F. Sun , et al. 2014. “Effects of Age and Sex on the Hematology and Blood Chemistry of Tibetan Macaques (*Macaca thibetana*).” Journal of the American Association for Laboratory Animal Science: JAALAS 53, no. 1: 12–17. 24411774 PMC3894642

[ajp70103-bib-0109] Xie, L. , F. Xu , S. Liu , et al. 2013. “Age‐and Sex‐Based Hematological and Biochemical Parameters for *Macaca fascicularis* .” PLoS One 8, no. 6: e64892. 10.1371/journal.pone.0064892. 23762263 PMC3677909

[ajp70103-bib-0110] Yamagata, K. , K. Ishida , T. Sairenchi , et al. 2007. “Risk Factors for Chronic Kidney Disease in a Community‐Based Population: A 10‐year Follow‐up Study.” Kidney International 71, no. 2: 159–166. 10.1038/sj.ki.5002017. 17136030

[ajp70103-bib-0111] Yamashita, N. 2008. “Chemical Properties of the Diets of Two Lemur Species in Southwestern Madagascar.” International Journal of Primatology 29: 339–364. 10.1007/s10764-008-9232-2.

[ajp70103-bib-0112] Zehr, S. M. , R. G. Roach , D. Haring , J. Taylor , F. H. Cameron , and A. D. Yoder . 2014. “Life History Profiles for 27 Strepsirrhine Primate Taxa Generated Using Captive Data From the Duke Lemur Center.” Scientific Data 1: 140019. 10.1038/sdata.2014.19. 25977776 PMC4322587

[ajp70103-bib-0113] Zucker, I. , and A. K. Beery . 2010. “Males Still Dominate Animal Studies.” Nature 465, no. 7299: 690. 10.1038/465690a. 20535186

