# Supplementary material for: Health Correlates of Extended Longevity in Captive Ring‐Tailed Lemurs (Lemur catta)
Source: Am J Primatol. 2025 Dec 11;87(12):e70103. doi: 10.1002/ajp.70103 (PMC12696616; doi:10.1002/ajp.70103)
Supplement: Supplementary file 1 — Mustill‐et‐al‐supporting‐information. [file AJP-87-e70103-s003.pdf]

Supporting information

Health correlates of extended longevity in captive ring-tailed lemurs (*Lemur catta*)

Ruby L. Mustill, Laura N. Ellsaesser, Cathy V. Williams, Megan Petersdorf, and Lydia K. Greene

<u>Index</u>

Table S1. Outlying datapoints excluded from the descriptive analyses and models reported in Tables 1 and 2.

Table S2. Summary statistics calculated including the outlying datapoints that were excluded from the descriptive analyses and models reported in Tables 1 and 2.

Table S3. Distribution families and link functions used in models of each serum chemistry analyte.

Table S4. Distribution families and link functions used in models of each CBC analyte.

Table S5. Fixed effect estimates and standard errors from models of each serum chemistry analyte. Estimates and standard errors are reported on the scale of the link function used in each model. Females are the reference group for the sex variable. Negative estimates under the "Sex" column indicate that predicted analyte values for males are lower than those for females.

Table S6. Fixed effect estimates and standard errors from models of each CBC analyte. Estimates and standard errors are reported on the scale of the link function used in each model. Females are the reference group for the sex variable. Negative estimates under the "Sex" column indicate that predicted analyte values for males are lower than those for females.

Table S7. Sex-specific effects of age on serum concentrations of blood urea nitrogen (BUN), albumin, creatine kinase, and cholesterol (i.e., the four markers that showed significant age-by-sex interactions in our main models). Estimates and standard errors are reported on the scale of the link function used in each model. Statistically significant results ($p \leq 0.05$) are in bold.

Table S8. Serum chemistry values (mean ± SD) in lemurs from the Duke Lemur Center, calculated using the following age groups: juvenile (< 5 years), adult (5–19.99 years), and geriatric adult (≥ 20 years). Statistics are reported with outlying datapoints excluded.

Table S9. CBC values (mean ± SD) in lemurs from the Duke Lemur Center, calculated using the following age groups: juvenile (< 5 years), adult (5–19.99 years), and geriatric adult (≥ 20 years).

Table S10. Serum chemistry values (mean ± SD) in lemurs from the Duke Lemur Center and from the Bezà Mahafaly Special Reserve, calculated using the same age groups (Singleton *et al.*, 2018). Duke Lemur Center statistics are reported with outlying datapoints excluded.

Table S11. CBC values (mean ± SD) in lemurs from the Duke Lemur Center and from the Bezà Mahafaly Special Reserve, calculated using the same age groups (Singleton *et al.*, 2018).

Table S12. Serum chemistry values (mean ± SD) in lemurs from the Duke Lemur Center and from St. Catherines Island, calculated using the same age groups (Page *et al.*, 2024). Duke Lemur Center statistics are reported with outlying datapoints excluded.

Table S13. CBC values (mean ± SD) in lemurs from the Duke Lemur Center and from St. Catherines Island, calculated using the same age groups (Page *et al.*, 2024).

Table S1. Outlying datapoints excluded from the descriptive analyses and models reported in Tables 1 and 2.

Analyte	Number of datapoints excluded	Value or range of excluded datapoint(s)	Range of included values	Sex and age of individual	Known health conditions of individual	Notes
Blood urea nitrogen (BUN; mg/dL)	1	75	8–52	Female; 18.12 years	None	We suspect this number is biologically real, but we removed it to be conservative.
Lipase (U/L)	6	756–1521	8–476	Male; 20.44–28.15 years	Pancreatic mass	All outliers are from one individual lemur.
Gamma-glutamyl transferase (GGT; U/L)	1	175	6–112	Female; 16.90 years	None	We suspect this number is biologically real, but we removed it to be conservative.
Alanine transaminase (ALT; U/L)	1	1117	27–562	Female; 3.98 years	None	We suspect this is an error in lab data entry (i.e., a repeated digit). The real value likely would have been 117.
Aspartate transaminase (AST; U/L)	1	332	8–125	Male; 4.85 years	None	We suspect this is an error in lab data entry (i.e., a repeated digit). The real value likely would have been 32.
Creatine kinase (U/L)	1	11888	357–5500	Female; 1.03 years	None	We suspect this number is biologically real, but we removed it to be conservative.
Cholesterol (mg/dL)	6	149–217	43–131	Female; 23.26–32.82 years	Type 2 diabetes	All outliers are from one individual lemur.
Calcium (Ca; mg/dL)	1	92	7.9–11.1	Male; 23.01 years	None	We suspect this is an error in lab data entry (i.e., a missing decimal point). The real value likely would have been 9.2.

Table S2. Summary statistics calculated including the outlying datapoints that were excluded from the descriptive analyses and models reported in Tables 1 and 2.

Analyte	Units	Range	Mean ± SD
Blood urea nitrogen (BUN)	mg/dL	8–75	22.43 ± 8.61
Lipase	U/L	8–1521	101.48 ± 214.58
Gamma-glutamyl transferase (GGT)	U/L	6–175	25.63 ± 19.46
Alanine transaminase (ALT)	U/L	27–1117	110.14 ± 103.60
Aspartate transaminase (AST)	U/L	8–332	29.69 ± 28.69
Creatine kinase	U/L	357–11888	1235.51 ± 1108.36
Cholesterol	mg/dL	43–217	84.24 ± 25.57
Calcium (Ca)	mg/dL	7.9–92.0	9.92 ± 6.37

Table S3. Distribution families and link functions used in models of each serum chemistry analyte.

Analyte	Distribution family	Link function
Blood urea nitrogen (BUN)	Gamma	Log
Creatinine	Gamma	Log
Amylase	Gaussian	Identity
Lipase	Gamma	Log
Gamma-glutamyl transferase (GGT)	Gamma	Log
Total bilirubin	Gamma	Log
Alkaline phosphatase	Gamma	Log
Alanine transaminase (ALT)	Gamma	Log
Aspartate transaminase (AST)	Gamma	Log
Albumin	Gamma	Log
Globulin	Gaussian	Identity
Creatine kinase	Negative binomial	Log
Cholesterol	Gaussian	Identity
Glucose	Gamma	Log
Sodium (Na)	Gaussian	Identity
Potassium (K)	Gamma	Log
Chloride (Cl)	Gaussian	Identity
Calcium (Ca)	Gamma	Log
Phosphate	Gamma	Log
Total CO_2	Gaussian	Identity
Anion gap	Gamma	Log

Table S4. Distribution families and link functions used in models of each CBC analyte.

Analyte	Distribution family	Link function
Red blood cell count	Gamma	Log
Hemoglobin (HGB)	Gaussian	Identity
Hematocrit (HCT)	Gaussian	Identity
White blood cell count	Negative binomial	Log
Lymphocytes	Gaussian	Identity
Monocytes	Gaussian	Identity
Neutrophils	Gaussian	Identity
Eosinophils	Gaussian	Identity

Table S5. Fixed effect estimates and standard errors from models of each serum chemistry analyte. Estimates and standard errors are reported on the scale of the link function used in each model. Females are the reference group for the sex variable. Negative estimates under the "Sex" column indicate that predicted analyte values for males are lower than those for females.

			Age		Sex (male)		Age * Sex	
Analyte	Distribution (link)	Final model terms	Estimate	SE	Estimate	SE	Estimate	SE
Blood urea nitrogen (BUN)	Gamma (log)	age * sex	0.016	0.005	0.130	0.100	-0.020	0.007
Creatinine	Gamma (log)	age + sex	0.003	0.002	-0.027	0.044		
Amylase	Gaussian (identity)	age + sex	38.777	7.185	-117.510	137.130		
Lipase	Gamma (log)	age + sex	0.030	0.009	0.018	0.160		
Gamma-glutamyl transferase (GGT)	Gamma (log)	age + sex	0.027	0.005	-0.266	0.097		
Total bilirubin	Gamma (log)	age + sex	-0.009	0.003	0.065	0.048		
Alkaline phosphatase	Gamma (log)	age + sex	0.003	0.005	-0.212	0.098		
Alanine transaminase (ALT)	Gamma (log)	age + sex	0.007	0.006	-0.193	0.113		
Aspartate transaminase (AST)	Gamma (log)	age + sex	-0.004	0.005	-0.169	0.100		
Albumin	Gamma (log)	age * sex	-0.002	0.001	0.055	0.025	-0.004	0.002
Globulin	Gaussian (identity)	age + sex	0.016	0.003	-0.089	0.062		
Creatine kinase	Negative binomial (log)	age * sex	-0.031	0.007	-0.413	0.151	0.029	0.010
Cholesterol	Gaussian (identity)	age * sex	0.501	0.263	3.656	5.418	-1.214	0.381
Glucose	Gamma (log)	age + sex	-0.001	0.003	-0.013	0.059		
Sodium (Na)	Gaussian (identity)	age + sex	0.041	0.027	-0.058	0.496		
Potassium (K)	Gamma (log)	age + sex	0.001	0.001	-0.028	0.024		
Chloride (Cl)	Gaussian (identity)	age + sex	-0.005	0.025	-0.081	0.445		
Calcium (Ca)	Gamma (log)	age + sex	-0.002	0.001	-0.004	0.010		
Phosphate	Gamma (log)	age + sex	-0.003	0.003	-0.154	0.059		
Total CO_2	Gaussian (identity)	age + sex	0.167	0.039	-0.262	0.690		
Anion gap	Gamma (log)	age + sex	-0.005	0.002	-0.012	0.035		

Table S6. Fixed effect estimates and standard errors from models of each CBC analyte. Estimates and standard errors are reported on the scale of the link function used in each model. Females are the reference group for the sex variable. Negative estimates under the "Sex" column indicate that predicted analyte values for males are lower than those for females.

			Age		Sex (male)	
Analyte	Distribution (link)	Final model terms	Estimate	SE	Estimate	SE
Red blood cell count	Gamma (log)	age + sex	0.004	0.001	0.034	0.022
Hemoglobin	Gaussian (identity)	age + sex	0.051	0.017	0.413	0.328
Hematocrit	Gaussian (identity)	age + sex	-0.089	0.082	2.903	1.411
White blood cell count	Negative binomial (log)	age + sex	-0.008	0.004	-0.167	0.070
Lymphocytes	Gaussian (identity)	age + sex	-0.068	0.188	2.512	3.446
Monocytes	Gaussian (identity)	age + sex	-0.012	0.020	-0.142	0.366
Neutrophils	Gaussian (identity)	age + sex	0.011	0.192	-2.582	3.494
Eosinophils	Gaussian (identity)	age + sex	0.036	0.028	0.124	0.512

Table S7. Sex-specific effects of age on serum concentrations of blood urea nitrogen (BUN), albumin, creatine kinase, and cholesterol (i.e., the four markers that showed significant age-by-sex interactions in our main models). Estimates and standard errors are reported on the scale of the link function used in each model. Statistically significant results ($p \leq 0.05$) are in bold.

		Age (females)				Age (males)			
Analyte	Distribution (link)	Estimate	SE	*z*	*p*	Estimate	SE	*z*	*p*
Blood urea nitrogen (BUN)	Gamma (log)	0.017	0.005	3.54	**<0.001**	-0.004	0.005	-0.78	0.44
Albumin	Gamma (log)	-0.002	0.001	-1.24	0.21	-0.006	0.001	-5.56	**<0.001**
Creatine kinase	Negative binomial (log)	-0.032	0.007	-4.25	**<0.001**	-0.002	0.007	-0.35	0.73
Cholesterol	Gaussian (identity)	0.557	0.250	2.23	**0.026**	-0.763	0.312	-2.45	**0.014**

Table S8. Serum chemistry values (mean ± SD) in lemurs from the Duke Lemur Center, calculated using the following age groups: juvenile (< 5 years), adult (5–19.99 years), and geriatric adult (≥ 20 years). Statistics are reported with outlying datapoints excluded.

		Duke Lemur Center lemurs		
Analyte	Units	Juvenile (< 5) *n* = 30	Adult (5–19.99) *n* = 39	Geriatric (≥ 20) *n* = 10
Blood urea nitrogen (BUN)	mg/dL	21.19 ± 3.81	21.57 ± 6.48	24.49 ± 11.93
Creatinine	mg/dL	0.75 ± 0.13	0.81 ± 0.16	0.85 ± 0.23
Amylase	U/L	1982.03 ± 382.50	2329.19 ± 521.63	2329.43 ± 685.61
Lipase	U/L	41.65 ± 22.72	52.74 ± 54.66	120.97 ± 104.67
Gamma-glutamyl transferase (GGT)	U/L	19.69 ± 7.92	24.38 ± 15.59	31.35 ± 20.07
Total bilirubin	mg/dL	0.53 ± 0.11	0.47 ± 0.11	0.41 ± 0.13
Alkaline phosphatase	U/L	208.50 ± 93.42	174.50 ± 74.75	166.38 ± 52.20
Alanine transaminase (ALT)	U/L	94.34 ± 49.17	107.63 ± 70.77	106.54 ± 80.90
Aspartate transaminase (AST)	U/L	33.02 ± 18.39	25.38 ± 15.00	28.32 ± 17.63
Albumin	g/dL	5.26 ± 0.39	5.29 ± 0.34	4.74 ± 0.52
Globulin	g/dL	1.14 ± 0.35	1.34 ± 0.30	1.54 ± 0.31
Creatine kinase	U/L	1716.73 ± 1109.21	931.96 ± 390.60	1152.73 ± 579.06
Cholesterol	mg/dL	81.60 ± 15.63	81.29 ± 17.53	78.19 ± 21.79
Glucose	mg/dL	184.83 ± 59.11	194.99 ± 73.69	180.78 ± 71.63
Sodium (Na)	mmol/L	144.98 ± 2.30	145.60 ± 2.83	146.43 ± 2.24
Potassium (K)	mmol/L	3.83 ± 0.30	4.06 ± 0.47	4.04 ± 0.46
Chloride (Cl)	mmol/L	108.69 ± 2.21	108.74 ± 3.11	109.00 ± 2.37
Calcium (Ca)	mg/dL	9.64 ± 0.47	9.45 ± 0.51	9.16 ± 0.54
Phosphate	mg/dL	4.97 ± 1.66	3.64 ± 1.04	4.27 ± 1.13
Total CO_2	mmol/L	20.81 ± 4.26	22.52 ± 3.75	24.95 ± 2.74
Anion gap	mmol/L	19.40 ± 4.35	18.50 ± 3.98	16.59 ± 3.48

Table S9. CBC values (mean ± SD) in lemurs from the Duke Lemur Center, calculated using the following age groups: juvenile (< 5 years), adult (5–19.99 years), and geriatric adult (≥ 20 years).

		Duke Lemur Center lemurs		
Analyte	Units	Juvenile (< 5)<br>*n* = 31	Adult (5–19.99)<br>*n* = 39	Geriatric (≥ 20)<br>*n* = 10
Red blood cell count	10^6 cells/µL	7.08 ± 0.58	7.49 ± 0.82	7.66 ± 0.88
Hemoglobin	g/dL	14.08 ± 1.38	14.93 ± 1.48	15.11 ± 1.67
Hematocrit	%	53.11 ± 4.84	54.54 ± 6.93	55.02 ± 5.64
White blood cell count	cells/µL	8853.49 ± 2811.06	7123.86 ± 2201.10	6832.43 ± 2214.73
Lymphocytes	%	48.03 ± 18.11	41.59 ± 16.13	41.99 ± 13.24
Monocytes	%	3.71 ± 2.42	3.82 ± 2.17	3.40 ± 2.30
Neutrophils	%	46.08 ± 18.64	51.42 ± 16.26	51.22 ± 13.27
Eosinophils	%	2.16 ± 2.60	3.10 ± 3.18	3.03 ± 3.62

Table S10. Serum chemistry values (mean ± SD) in lemurs from the Duke Lemur Center and from the Bezà Mahafaly Special Reserve, calculated using the same age groups (Singleton *et al.*, 2018). Duke Lemur Center statistics are reported with outlying datapoints excluded.

		Duke Lemur Center lemurs			Bezà Mahafaly lemurs (Singleton *et al.*, 2018)		
Analyte	Units	Young (< 5) *n* = 30	Adult (5–9.99) *n* = 30	Old (≥ 10) *n* = 25	Young (< 5) *n* = 8	Adult (5–9.99) *n* = 18	Old (≥ 10) *n* = 18
Blood urea nitrogen (BUN)	mg/dL	21.19 ± 3.81	20.98 ± 5.10	23.15 ± 9.72	7 ± 5	9 ± 6	12 ± 10
Creatinine	mg/dL	0.75 ± 0.13	0.81 ± 0.14	0.83 ± 0.20	0.9 ± 0.2	0.8 ± 0.1	1.0 ± 0.2
Amylase	U/L	1982.03 ± 382.50	2180.15 ± 434.40	2401.12 ± 618.54		*not measured*	
Lipase	U/L	41.65 ± 22.72	43.88 ± 37.56	84.82 ± 88.07		*not measured*	
Gamma-glutamyl transferase (GGT)	U/L	19.69 ± 7.92	19.17 ± 8.04	30.06 ± 19.38		*not measured*	
Total bilirubin	mg/dL	0.53 ± 0.11	0.48 ± 0.11	0.45 ± 0.12		*not measured*	
Alkaline phosphatase	U/L	208.50 ± 93.42	154.50 ± 63.02	180.85 ± 69.70		*not measured*	
Alanine transaminase (ALT)	U/L	94.34 ± 49.17	90.79 ± 38.48	115.48 ± 84.77		*not measured*	
Aspartate transaminase (AST)	U/L	33.02 ± 18.39	22.38 ± 8.54	28.14 ± 18.10		*not measured*	
Albumin	g/dL	5.26 ± 0.39	5.27 ± 0.31	5.05 ± 0.52		*not measured*	
Globulin	g/dL	1.14 ± 0.35	1.30 ± 0.24	1.44 ± 0.33		*not measured*	
Creatine kinase	U/L	1716.73 ± 1109.21	952.14 ± 455.06	1018.08 ± 466.94		*not measured*	
Cholesterol	mg/dL	81.60 ± 15.63	79.48 ± 17.54	81.04 ± 19.33		*not measured*	
Glucose	mg/dL	184.83 ± 59.11	198.40 ± 72.18	187.12 ± 73.67	209 ± 109	162 ± 84	152 ± 57
Sodium (Na)	mmol/L	144.98 ± 2.30	145.98 ± 2.97	145.78 ± 2.56	143 ± 3	143 ± 3	142 ± 3
Potassium (K)	mmol/L	3.83 ± 0.30	3.97 ± 0.51	4.10 ± 0.44	4.2 ± 0.7	3.6 ± 0.5	3.5 ± 0.4
Chloride (Cl)	mmol/L	108.69 ± 2.21	109.45 ± 3.22	108.51 ± 2.70	103 ± 4	105 ± 5	104 ± 4
Calcium (Ca)	mg/dL	9.64 ± 0.47	9.48 ± 0.48	9.31 ± 0.55		*not measured*	
Phosphate	mg/dL	4.97 ± 1.66	3.39 ± 1.06	4.04 ± 1.06		*not measured*	
Total CO_2	mmol/L	20.81 ± 4.26	22.50 ± 3.92	23.59 ± 3.47	26 ± 4	23 ± 5	26 ± 6
Anion gap	mmol/L	19.40 ± 4.35	18.10 ± 4.09	17.87 ± 3.87	19 ± 1	19 ± 3	18 ± 2

Table S11. CBC values (mean ± SD) in lemurs from the Duke Lemur Center and from the Bezà Mahafaly Special Reserve, calculated using the same age groups (Singleton *et al.*, 2018).

		Duke Lemur Center lemurs			Bezà Mahafaly lemurs (Singleton *et al.*, 2018)		
Analyte	Units	Young (< 5) *n* = 31	Adult (5–9.99) *n* = 30	Old (≥ 10) *n* = 25	Young (< 5) *n* = 8	Adult (5–9.99) *n* = 18	Old (≥ 10) *n* = 18
Red blood cell count	10^6 cells/µL	7.08 ± 0.58	7.29 ± 0.75	7.66 ± 0.85		*not measured*	
Hemoglobin	g/dL	14.08 ± 1.38	14.59 ± 1.38	15.18 ± 1.58	12.5 ± 1.4	12.2 ± 1.0	11.6 ± 1.0
Hematocrit	%	53.11 ± 4.84	53.75 ± 6.23	55.15 ± 6.70	36.0 ± 4.0	35.8 ± 3.0	33.9 ± 2.9
White blood cell count	cells/µL	8853.49 ± 2811.06	7107.14 ± 2318.16	7002.41 ± 2151.74	8669 ± 2517	8291 ± 2230	6273 ± 1912
Lymphocytes	%	48.03 ± 18.11	40.46 ± 19.00	42.35 ± 13.09	58.5 ± 13.6	55.4 ± 12.1	43.3 ± 11.7
Monocytes	%	3.71 ± 2.42	3.82 ± 2.01	3.63 ± 2.31	7.6 ± 2.9	4.9 ± 1.9	6.1 ± 3.0
Neutrophils	%	46.08 ± 18.64	52.90 ± 19.46	50.58 ± 12.91	27.5 ± 12.7	33.4 ± 11.9	43.2 ± 10.3
Eosinophils	%	2.16 ± 2.60	2.80 ± 2.93	3.22 ± 3.49	4.0 ± 3.4	4.6 ± 3.3	5.4 ± 3.5

Table S12. Serum chemistry values (mean ± SD) in lemurs from the Duke Lemur Center and from St. Catherines Island, calculated using the same age groups (Page *et al.*, 2024). Duke Lemur Center statistics are reported with outlying datapoints excluded.

		Duke Lemur Center lemurs			St. Catherines Island lemurs (Page *et al.*, 2024)		
Analyte	Units	Infant (< 1) *n* = 7	Juvenile (1–5.99) *n* = 35	Adult (≥ 6) *n* = 35	Infant (< 1) *n* = 29	Juvenile (1–5.99) *n* = 36	Adult (≥ 6) *n* = 20
Blood urea nitrogen (BUN)	mg/dL	22.43 ± 5.22	21.69 ± 4.21	22.30 ± 8.92	19.4 ± 5.6	25.7 ± 6.4	27.3 ± 7.5
Creatinine	mg/dL	0.69 ± 0.13	0.77 ± 0.13	0.83 ± 0.19	0.9 ± 0.2	1.1 ± 0.2	1.2 ± 0.3
Amylase	U/L	1746.00 ± 382.02	2083.58 ± 363.58	2347.65 ± 597.31	1627 ± 356	1574 ± 322	1551 ± 375
Lipase	U/L	27.43 ± 9.90	43.23 ± 28.66	75.34 ± 80.34	36.6 ± 20.9	63.5 ± 30.3	73.8 ± 26.9
Gamma-glutamyl transferase (GGT)	U/L	18.86 ± 7.08	19.92 ± 7.87	27.42 ± 18.12	10.7 ± 5.2	20.5 ± 8.9	22.2 ± 7.2
Total bilirubin	mg/dL	0.56 ± 0.10	0.52 ± 0.11	0.45 ± 0.12	0.4 ± 0.1	0.5 ± 0.2	0.5 ± 0.2
Alkaline phosphatase	U/L	263.43 ± 38.36	185.65 ± 87.94	173.82 ± 69.89	402.7 ± 132.1	206.3 ± 77.0	199.7 ± 77.4
Alanine transaminase (ALT)	U/L	86.57 ± 19.28	95.35 ± 51.95	109.35 ± 76.45	55.6 ± 16.6	80.1 ± 42.2	66.3 ± 25.7
Aspartate transaminase (AST)	U/L	40.57 ± 22.87	28.86 ± 15.28	26.64 ± 16.65	38 ± 17.1	31.4 ± 28.4	24.7 ± 12.3
Albumin	g/dL	5.16 ± 0.42	5.29 ± 0.35	5.10 ± 0.49	5.4 ± 0.8	5.8 ± 0.5	5.7 ± 0.5
Globulin	g/dL	0.96 ± 0.23	1.22 ± 0.34	1.41 ± 0.32	0.9 ± 0.4	1.3 ± 0.5	1.6 ± 0.5
Creatine kinase	U/L	2150.29 ± 906.02	1421.22 ± 1012.53	994.35 ± 456.40	4205 ± 2615	2207 ± 4275	1173 ± 624
Cholesterol	mg/dL	86.86 ± 9.19	81.33 ± 16.30	80.10 ± 19.08	131.0 ± 26.3	98.6 ± 20.8	103.3 ± 19.6
Glucose	mg/dL	204.14 ± 48.63	179.85 ± 68.81	192.91 ± 71.31	269.9 ± 85.6	176.1 ± 59.1	168.6 ± 58.2
Sodium (Na)	mmol/L	143.86 ± 2.79	145.54 ± 2.78	145.78 ± 2.51	147.4 ± 10.4	151.4 ± 11.4	148.3 ± 3.1
Potassium (K)	mmol/L	3.91 ± 0.41	3.79 ± 0.32	4.10 ± 0.46	5.0 ± 0.7	4.1 ± 0.6	4.1 ± 0.3
Chloride (Cl)	mmol/L	108.00 ± 2.00	108.98 ± 2.88	108.75 ± 2.73	106.2 ± 7.9	109.1 ± 5.8	107.9 ± 3.6
Calcium (Ca)	mg/dL	9.93 ± 0.65	9.54 ± 0.42	9.35 ± 0.54	10.7 ± 2.0	9.9 ± 0.7	9.7 ± 0.6
Phosphate	mg/dL	6.03 ± 1.60	4.35 ± 1.55	3.88 ± 1.11	10.3 ± 2.0	6.7 ± 1.7	5.4 ± 1.8
Total CO_2	mmol/L	14.71 ± 3.09	21.96 ± 3.42	23.45 ± 3.60		*not measured*	
Anion gap	mmol/L	25.00 ± 2.65	18.52 ± 3.53	17.78 ± 4.02		*not measured*	

Table S13. CBC values (mean ± SD) in lemurs from the Duke Lemur Center and from St. Catherines Island, calculated using the same age groups (Page *et al*., 2024).

		Duke Lemur Center lemurs			St. Catherines Island lemurs (Page *et al*., 2024)		
Analyte	Units	Infant (< 1) *n* = 7	Juvenile (1–5.99) *n* = 36	Adult (≥ 6) *n* = 35	Infant (< 1) *n* = 29	Juvenile (1–5.99) *n* = 36	Adult (≥ 6) *n* = 20
Red blood cell count	10^6 cells/µL	6.90 ± 0.42	7.13 ± 0.61	7.59 ± 0.85	7.1 ± 0.7	7.2 ± 0.8	7.1 ± 0.6
Hemoglobin	g/dL	13.36 ± 0.76	14.25 ± 1.38	15.09 ± 1.55	13.7 ± 1.2	14.8 ± 1.4	14.6 ± 1.2
Hematocrit	%	51.33 ± 3.46	52.96 ± 5.45	55.12 ± 6.51	43.1 ± 5.5	45.8 ± 4.9	43.6 ± 5.5
White blood cell count	cells/µL	9042.86 ± 4460.51	8322.64 ± 2344.52	7000.00 ± 2273.25	6300 ± 3100	6100 ± 2200	7100 ± 3300
Lymphocytes	%	46.57 ± 17.65	45.01 ± 18.81	42.29 ± 14.79	46.7 ± 15.7	40.1 ± 16.3	33.9 ± 11.8
Monocytes	%	2.57 ± 0.79	3.84 ± 2.39	3.70 ± 2.24	3.4 ± 2.7	3.8 ± 3.2	4.0 ± 2.7
Neutrophils	%	49.71 ± 18.27	48.83 ± 19.12	50.61 ± 14.88	48.4 ± 16.2	54.6 ± 15.9	59.8 ± 13.5
Eosinophils	%	1.14 ± 1.21	2.30 ± 2.53	3.22 ± 3.45	1.5 ± 2.7	1.5 ± 2.5	2.3 ± 3.0

References

Page, A., Brenner, D., & Norton, T. M. (2024). Retrospective hematology and serum biochemistry of ring-tailed lemurs (*Lemur catta*) on St. Catherines Island, Georgia, USA. *Journal of Zoo and Wildlife Medicine*, *55*(2), 436–446. https://doi.org/10.1638/2022-0088.

Singleton, C. L., Sauther, M. L., Cuozzo, F. P., & Jacky, I. A. Y. (2018). Age-related changes in hematology and blood biochemistry values in endangered, wild ring-tailed lemurs (*Lemur catta*) at the Bezà Mahafaly Special Reserve, Madagascar. *Journal of Zoo and Wildlife Medicine*, *49*(1), 30–47. https://doi.org/10.1638/2017-0008R1.1.
